# Discovery of Potential Scaffolds for Methionine Adenosyltransferase 2A (MAT2A) Inhibitors: Virtual Screening, Synthesis, and Biological Evaluation

**DOI:** 10.3390/molecules30102134

**Published:** 2025-05-12

**Authors:** Chunchun Qi, Xinghui Yu, Siyu Zuo, Pinsheng Han, Ruonan An, Yamin Zhang

**Affiliations:** 1 School of Medicine, Nankai University, Tianjin 300071, China; qwen2708@163.com (C.Q.); 1120210743@mail.nankai.edu.cn (X.Y.); zuosymailbox@163.com (S.Z.); 1120230903@mail.nankai.edu.cn (P.H.); anruonan0207@outlook.com (R.A.); 2 Department of Hepatobiliary Surgery, Tianjin First Central Hospital, School of Medicine, Nankai University, Tianjin 300071, China

**Keywords:** methionine adenosyltransferase 2A (MAT2A), synthetic lethality, virtual screening

## Abstract

The inhibition of methionine adenosyltransferase 2A (MAT2A) in cancers harboring deletions of the methylthioadenosine phosphorylase (MTAP) gene induces synthetic lethality, making it a highly compelling strategy in the pursuit of precision anticancer therapeutics. In this study, structure-based computing methods were employed to discover novel scaffolds as potential MAT2A inhibitors. The most potent compound, **17**, demonstrated inhibition of MAT2A with an IC_50_ of 0.43 μM, and showed antitumor effects against MTAP^−/−^ HCT116 cells with an IC_50_ of 1.4 μM. The identified compounds and their associated structural data could provide valuable insights for related drug discovery projects.

## 1. Introduction

Methionine adenosyltransferase 2A (MAT2A) is a crucial enzyme that catalyzes the synthesis of S-adenosylmethionine (SAM), the primary methyl donor involved in DNA, RNA, and protein methylation, as well as various metabolic pathways [1,2]. As one of the three methionine adenosyltransferase (MAT) isoforms, MAT2A is predominantly expressed in extrahepatic tissues and cancer cells, where it supports tumor growth by driving transcriptional and metabolic reprogramming [3]. Notably, MAT2A is essential for the survival of cancers harboring deletions of the methylthioadenosine phosphorylase (MTAP) gene, which occurs in approximately 13–15% of tumors due to its co-deletion with the CDKN2A tumor suppressor [4,5]. The loss of MTAP leads to the accumulation of 5′-methylthioadenosine (MTA), a metabolite that inhibits protein arginine methyltransferase 5 (PRMT5), thereby reducing its methyltransferase activity. Under these conditions, MTAP-deficient tumors become highly dependent on MAT2A to maintain intracellular SAM levels [6]. Inhibiting MAT2A further depletes SAM, exacerbating PRMT5 suppression and selectively impairing tumor cell proliferation while sparing normal tissues [7,8,9]. Given this synthetic lethal interaction, MAT2A has emerged as a promising therapeutic target, with several inhibitors currently in clinical development for the treatment of MTAP-deleted cancers [10,11].

**Figure 1:**
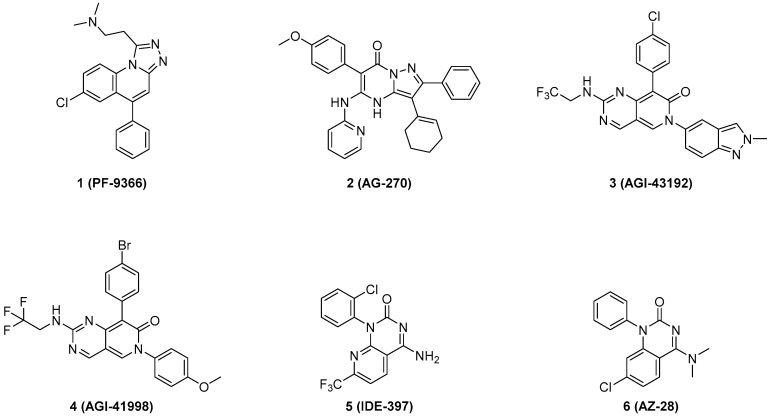
Representative small-molecule allosteric inhibitors of MAT2A.

As shown in Figure 1, the first small-molecule allosteric inhibitor of MAT2A, **1** (PF-9366) [12], was disclosed by Pfizer in 2017. However, treatment with PF-9366 upregulates MAT2A transcript and protein levels, thereby reducing its cellular potency. Agios Pharmaceuticals subsequently developed **2** (AG-270) [13], the first oral MAT2A inhibitor to reach clinical evaluation. Despite its promise, the Phase I trial for AG-270 was terminated in 2023 (NCT03435250). Agios also disclosed **3** (AGI-43192) and **4** (AGI-41998) [14] with limited brain penetration and peripherally efficacious properties. Compound **5** (IDE-397, also known as GSK-4362676) entered a phase 1 clinical trial in 2021 (NCT04794699) [15]. AstraZeneca also published a series of MAT2A inhibitors including **6** (AZ-28) [16], which exhibited excellent potency in vitro. However, achieving in vivo efficacy in mice required high doses and subcutaneous administration due to its high clearance. Recently, structure-based drug design efforts also led to the identification of MAT2A inhibitors with novel scaffolds and binding modes [17,18,19,20,21]. These extensive medicinal chemistry efforts underscore the growing interest in MAT2A as a therapeutic target.

In this study, a multistep virtual screening strategy was used to discover novel scaffolds as potential MAT2A inhibitors. Through a cascade of structure-based virtual screening and pharmacophore model filtering, 11 candidate molecules were cherry-picked for further inhibitory activity evaluation. Two of these molecules demonstrated moderate activities. The scaffold-hopping strategy was applied to further improve in vitro potency. This approach led to the identification of compound **17** as the most potent inhibitor of MAT2A, effectively suppressing the growth of MTAP^−/−^ HCT116 cells.

## 2. Results

### 2.1. Pharmacophore Modeling and Validation

Until this work was initiated, there were 35 MAT2A co-crystal structures containing allosteric inhibitors with a resolution of <2.5 Å released in the Protein Data Bank. We collected co-crystal structures of six MAT2A complexes to build pharmacophore models, including 7BHV [16], 7KCC [13], 7RWG [14], 8P4H [17], 8QE2 [21], and 8XB0 [20]. Schrödinger software was used to pretreat and optimize the MAT2A complex. Each of the protein–ligand complexes was prepared and aligned to 7KCC. Ligand-based pharmacophore models [22] were applied and a total of 10 pharmacophore models were generated (Figure S1).

**Figure 2:**
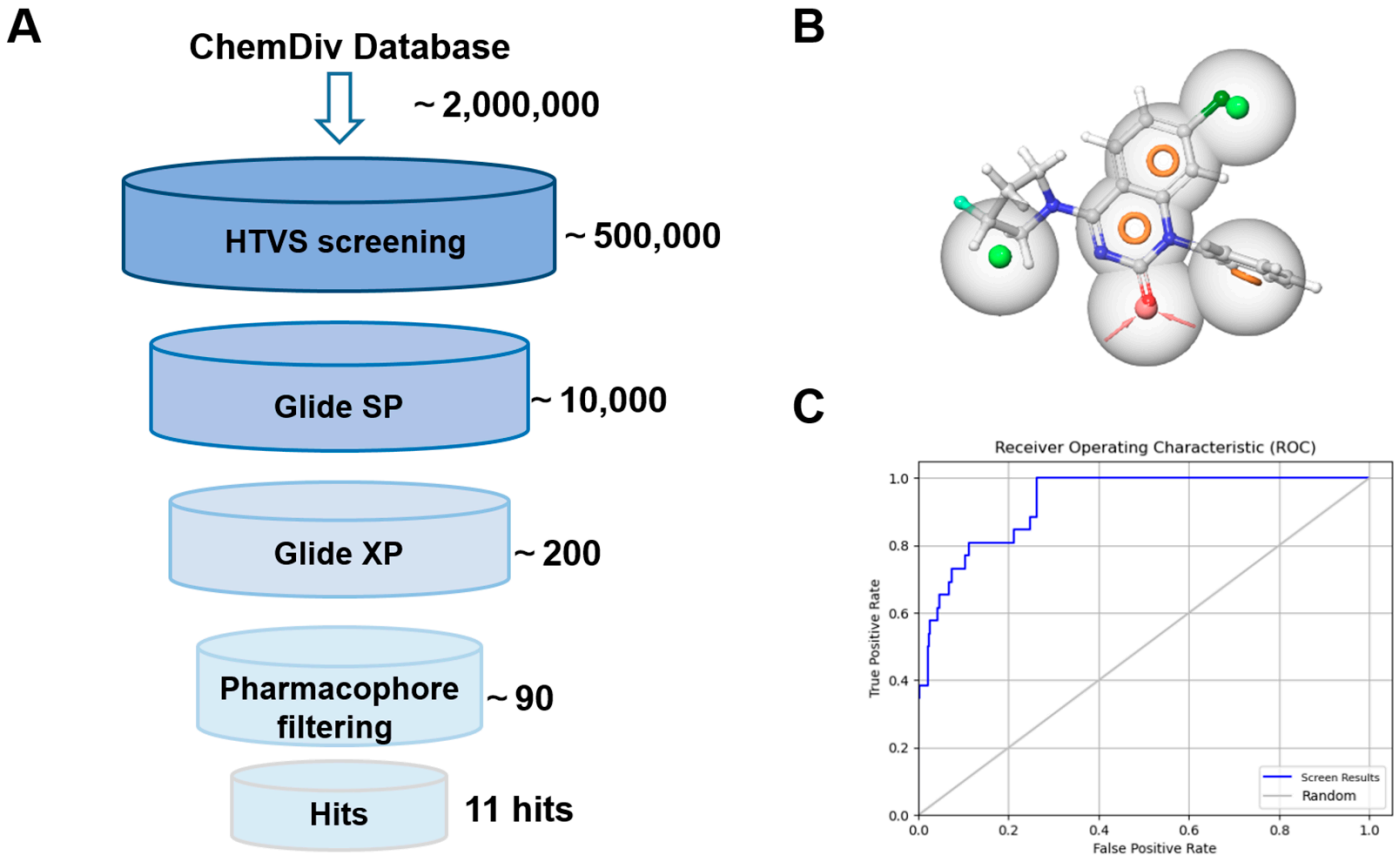
(**A**) Schematic flow of the virtual screening campaign. (**B**) The pharmacophore features of model 9. Green: hydrophobic groups; yellow: aromatic groups; red: acceptor atom. (**C**) The ROC curve of model 9.

For model validation, we organized a test dataset composed of 26 active compounds (see Table S1) and 1150 decoys generated by DUDE decoys database [23]. The quality of the pharmacophore model, specifically its ability to enrich active compounds from the dataset, was assessed by calculating enrichment factors (EFs) and receiver operating characteristic (ROC) curves (Table S2) [24]. Model 9 showed the best enrichment potency, with an EF1% value of 34.68% and a ROC value of 0.93 (Figure 2B). The different colors represent different types of constituents: two hydrophobic groups (green), three aromatic groups (yellow), and one acceptor atom (red). The sphere size indicates query tolerance.

### 2.2. Multistep Virtual Screening

**Figure 3:**
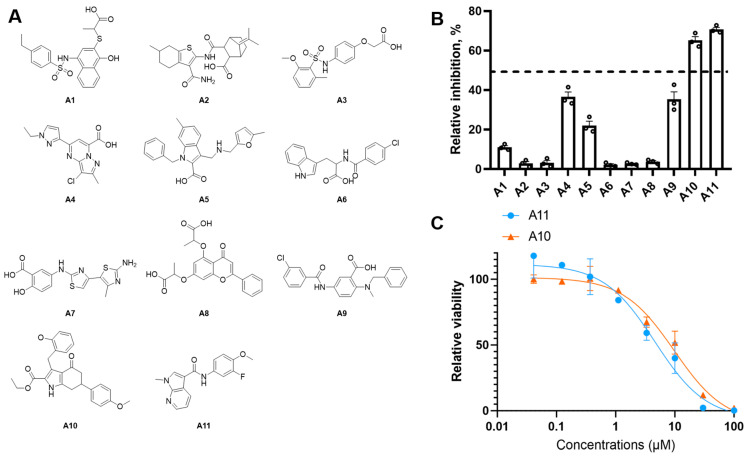
(**A**) Chemical structure of virtual hits **A1**–**A11**. (**B**) The antiproliferative effect of hit compounds on MAT2A was evaluated at 10 μM. The relative inhibition rate of each compound was determined in triplicate relative to the control group. Data are represented as mean ± SD (n = 3). (**C**) The dose–dependency curves of compounds **A10** and **A11** against MAT2A.

As shown in Figure 2A, the commercial database ChemDiv chemical library was docked into the allosteric region of model 7KCC [13] by an HTVS module. The top 5% of docking poses from the screened molecules, selected based on docking scores, were retained and subjected to the SP scoring function. The highest-ranking SP pose of each compound was then rescored using the XP scoring function in Glide, and the resulting poses were further filtered through a pharmacophore model. Through a cascade of drug-likeness filtering, HTVS docking, SP docking, XP docking, pharmacophore filtering, and visual inspection, 11 structurally diverse compounds were cherry-picked and purchased from the ChemDiv chemical library (Figure 3A). These compounds were evaluated by enzymatic assay. Initial inhibitory activity was conducted for each compound at concentrations of 10 μM (Figure 3B and Figure S3). Compounds **A10** and **A11** exhibited inhibitory potency at a lower concentration of 10 μM. We further measured the dose curves. As shown in Figure 3C, compounds **A10** and **A11** displayed moderate inhibitory activity with IC_50_ values of 6.8 and 4.6 μM, respectively.

**Figure 4:**
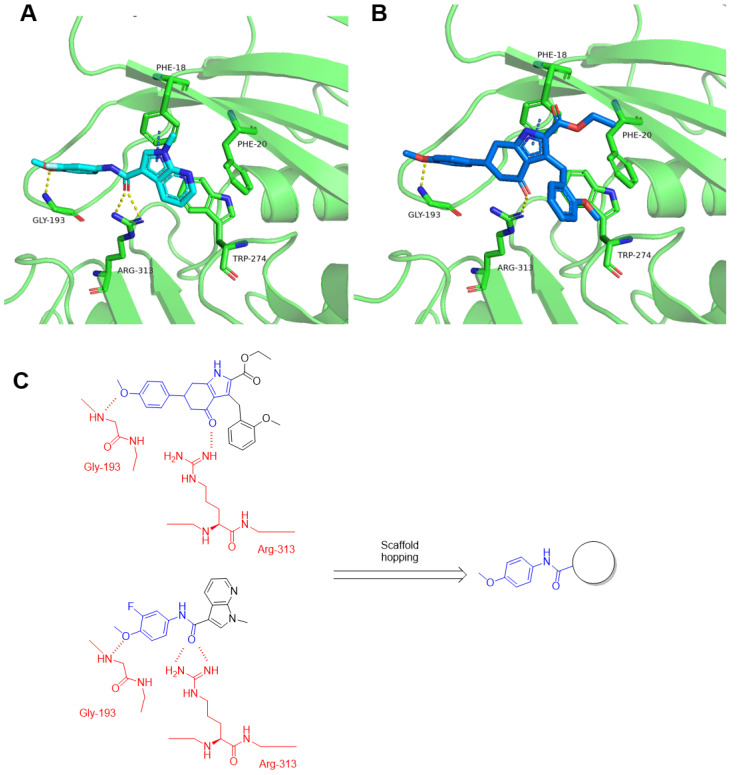
Predicted binding modes of compound **A10** (**A**) and compound **A11** (**B**) with MAT2A crystal structure (PDB id: 7KCC). Hydrogen bonds were indicated by yellow dash lines. (**C**) Design strategy of new MAT2A inhibitors. Red: key residues in the MAT2A protein; blue: pharmacophore features of the ligands.

As shown in Figure 4A and Figure S5, the docking study revealed that compound **A10** was well embedded within the allosteric binding site, forming a crucial hydrogen bond with Arg-313. Gly-193 established another key hydrogen bond with the methoxyphenyl moiety. Additionally, the pyrrole moiety was positioned within a hydrophobic cavity created by Phe-20 and Trp-274, further stabilizing the interaction. The binding mode of compound **A11** showed a similar pattern.

### 2.3. Structure–Activity Relationship (SAR) Study

Our initial efforts were built upon the **A10** scaffold as a structural foundation. As shown in Table 1, compound **A10** exhibited the strongest inhibitory activity (IC_50_ = 4.6 μM), followed by **8** (IC_50_ = 6.8 μM), whereas **9** and **10** showed no detectable activity at 10 μM. Despite losing the Gly-193 hydrogen bond due to the absence of a methoxy group, compound 8 maintains high activity, likely through compensatory hydrophobic interactions and optimized binding pose geometry within the pocket. The superior potency of **A10** suggests that its specific substitution pattern, particularly the methoxy functionality, plays a crucial role in enhancing MAT2A binding. The complete loss of activity in compounds **9** and **10** may be attributed to the loss of the hydrogen bond with Gly-193. The modification space of compound **A10** was limited, making it prone to adopting an energetically frustrated conformation. The low yield also led us to focus our further activity optimization efforts on the scaffold of **A11**. As compound **A10** aligned well with **A11** (Figure S4), we hypothesized that **A11** corresponded to the ring-opened form of **A10**. As shown in Figure 4C, the scaffold-hopping strategy was applied to further improve activity.

Guided by the binding mode hypothesis, we explored the structure–activity relationship of **A11** derivatives. As shown in Table 2, analog **11** exhibited a significant reduction in inhibitory activity, suggesting that hydrophobic interactions involving the R group play a crucial role in maintaining inhibition. To further investigate the chemical space, we introduced bicyclic systems. Compounds **12** and **13** demonstrated inhibitory potency comparable to **A11**, whereas compound **14** completely lost its binding affinity, indicating that the benzo[*d*][1,3]dioxole moiety may disrupt critical interactions with Gly-193. Encouragingly, compound **15** displayed a two-fold increase in activity compared to **A11**, with docking studies revealing a strong π-π interaction with Phe-18 (Figure 4A). In contrast, compound **16** exhibited a complete loss of binding affinity, likely due to steric hindrance and the adoption of a perpendicular conformation. To further exploit hydrophobic and π-π interactions, we replaced the benzothiazole scaffold with a biaryl group, yielding compound **17**. Compound **17** further enhanced MAT2A inhibitory activity, with an IC_50_ of 0.43 μM.

The cellular antiproliferation assay for several active compounds was conducted on HCT116 colon cancer cells (Table 3). Among this series, compounds **15** and **17** demonstrated enhanced cellular activity compared to **A11**, suggesting that the scaffold-hopping strategy successfully translated into improved in vitro potency.

**Figure 5:**
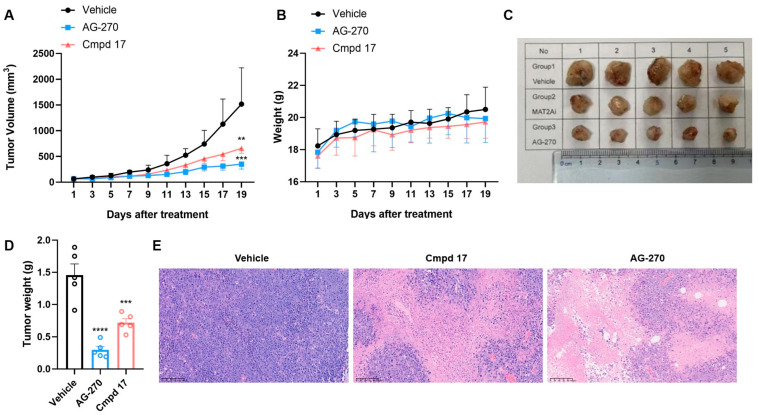
In vivo efficacy of compound **17** in an HCT116 MTAP-deleted xenograft tumor model in mice. (**A**) Tumor growth curve of three groups, including vehicle control, **17** (30 mg/kg, qd), and AG-270 (30 mg/kg, qd); (**B**) The body weights of the group mice over time; (**C**) Photographs of the isolated tumor tissues; (**D**) The tumor weight of three groups; data are plotted as the mean ± SD, and significance of differences was determined by one-way ANOVA. (**E**) Representative H&E staining figures of tumor tissues after treatment. (** *p* < 0.01, *** *p* < 0.001, **** *p* < 0.0001).

The in vivo antitumor efficacy of compound **17** was evaluated in a xenograft model using HCT116 MTAP-deleted cells (Figure 5). Seven days after inoculation, mice were randomly divided into three groups (n = 5). AG-270 served as the reference compound, and both agents were administered orally at 30 mpk once daily (q.d.) for 19 consecutive days. Compound **17** exhibited the significant tumor growth inhibition (TGI) of 58.4%, compared to 80.3% for AG-270 (Table S4). The dosage of **17** was well tolerated, with minimal impact on body weight and no observed mortality. The histopathological analysis (H&E staining) of tumor tissues revealed reduced cellular proliferation in both the **17**- and AG-270-treated groups.

**Figure 6:**
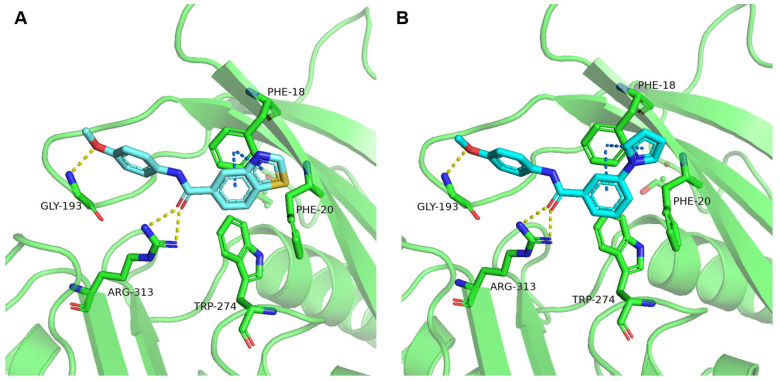
Predicted binding mode of compound **15** (**A**) and compound **17** (**B**) with MAT2A crystal structure (PDB id: 7KCC). Key residues of MAT2A are highlighted in green, with bound ligands displayed in cyan. Hydrogen bonds are indicated by yellow dash lines. Face-to-face π-π interactions are indicated by blue dash lines.

To elucidate the observed activities of **A11** derivatives against MAT2A, molecular modeling was conducted (Figure 6). The amide group formed a crucially strong hydrogen bond network with Arg-313, while Gly-193 established another key hydrogen bond with the methoxyphenyl moiety. Notably, a face-to-face π-π interaction was observed between the biaryl moiety and Phe-18. In accordance with the SAR of Table 2, aromatic substituents were well tolerated.

## 3. Experimental Section

### 3.1. General Information

All starting materials were commercially available and used without further purification. All reactions were carried out without purification unless otherwise stated. All reactions were monitored by thin-layer chromatography (TLC) carried out on silica gel plates, and components were visualized using UV light (254 nm or 365 nm) or iodine vapor. Solvent removal refers to rotary evaporation under reduced pressure at 40–45 °C. The ^1^H NMR and ^13^C NMR spectra were collected on a 400 MHz Bruker spectrometer (Bruker Corporation, San Jose, CA, USA). Flash column chromatography was performed using silica gel on a SepaBean^®^ machine U100 preparative liquid chromatography system (Santaitech, Changzhou, China). Purity was ascertained from AUC by HPLC using a Angilent 1260 system (G7111A Quat pump and G7114A VWD detector) with a Poroshell 120 EC-C18 reversed-phase column (4 μm) at 254 nM (Agilent Technologies Inc., Santa Clara, CA, USA). A linear gradient program using water (solvent A) and acetonitrile (solvent B); t = 0–2 min, 10% B, t = 18 min, 90% B, was employed. Flow rate was 1.2 mL/min and UV detection was set to 254 nm.

#### Chemistry

**Scheme 1:**
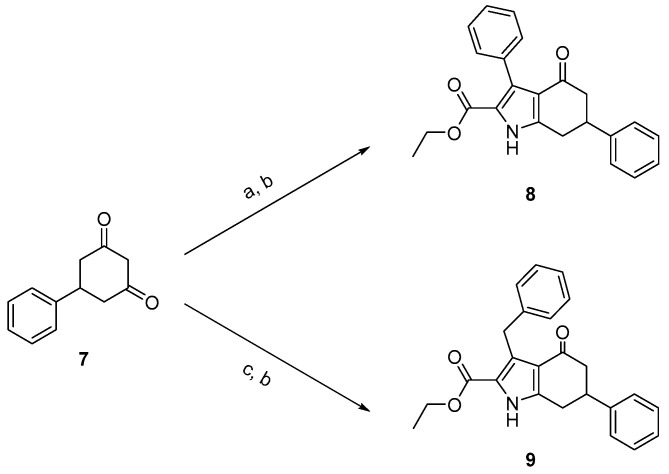
Synthesis of compounds **8** and **9**. Reagents and conditions: (**a**) ethyl acetylacetate, NaNO_2_, AcOH, 0 °C to r.t.; (**b**) zinc powder, 60 °C, yield 4–8%; (**c**) ethyl 3-oxo-4-phenylbutanoate, NaNO_2_, AcOH, 0 °C to r.t.

The synthesis of compounds **8** and **9** was performed according to Scheme 1. Compound **7** was treated with ethyl acetylacetate in acetic acid to create the intermediate, which was then reacted with zinc powder to create compound **8**. Compound **9** was prepared following a similar procedure.

**Scheme 2:**
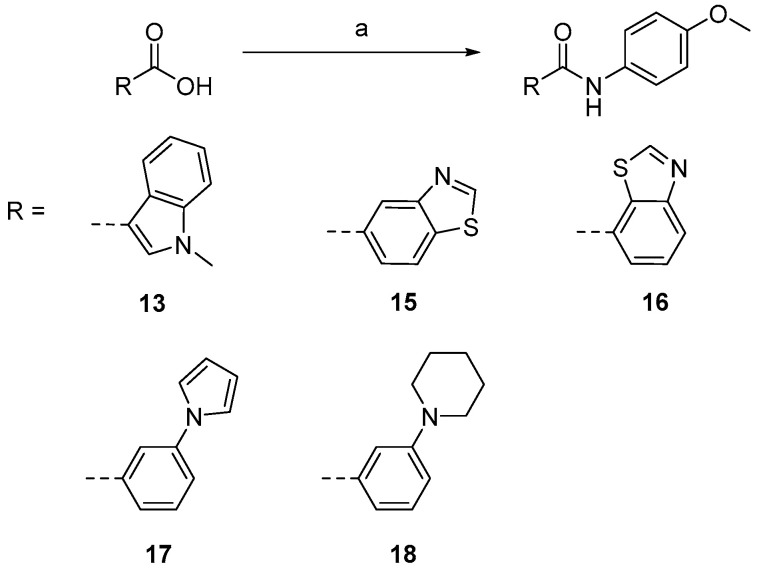
Synthesis of compounds **16**–**21**. Reagents and conditions [21]: (**a**) anisidine, BOP, DIPEA, DMF, 40 °C, yield 37–72%.

Compounds **16**–**21** were prepared according to Scheme 2. Commercially available acid was treated with BOP in DMF for 20 min, then anisidine was added. All of the compounds were checked by ^1^H NMR and ^13^C NMR.

### 3.2. General Procedure A for Synthesis of Compounds ***16***–***21***

To a solution of acid (1.2 eq) in DMF (3 mL), BOP (1.5 eq) and DIPEA (3 eq) were added. The reaction was stirred at 40 °C for 30 min, then anisidine (1.0 eq) was added. After the reaction was finished by TLC, the mixture was poured into water and extracted with ethyl acetate. The organic layer was dried over Na_2_SO_4_, filtered, and concentrated. The residue was purified by silica gel column chromatography (DCM/MeOH, 30:1) followed by recrystallization in acetonitrile to provide the desired product.

### 3.3. Ethyl 4-Oxo-3,6-diphenyl-4,5,6,7-tetrahydro-1H-indole-2-carboxylate (***8***)

NaNO_2_ (1.1 eq) and ethyl acetylacetate (1.1 eq) were added to a solution of **7** (1.0 eq) in acetic acid (3 mL) at 0 °C. The reaction was allowed to come to room temperature and was stirred for 2 h; then zinc powder (2.2 eq) was added. The mixture was heated to 80 °C with vigorous stirring. After the reaction was finished by TLC, the mixture was poured into water and extracted with ethyl acetate. The organic layer was dried over Na_2_SO_4_, filtered, and concentrated. The residue was purified by HPLC to produce compound **10** as a white solid (5.1 mg, yield 8%). ^1^H NMR (400 MHz, DMSO-*d*_6_) δ 12.33 (s, 1H, NH), 7.41–7.23 (m, 10H), 4.07 (q, *J* = 7.2 Hz, 2H, -OCH_2_-), 3.56–3.45 (m, 1H), 3.12–3.02 (m, 2H), 2.90–2.72 (m, 1H), 2.44–2.37 (m, 1H), and 1.04 (t, *J* = 7.2 Hz, 3H, -CH_3_). ^13^C NMR (101 MHz, DMSO-*d*_6_) δ 192.11, 160.88, 146.24, 144.09, 133.98, 130.92, 129.88, 129.00, 127.42, 127.17, 127.08, 119.81, 118.62, 117.97, 60.21, 46.49, 41.26, 30.57, and 14.33. LC-MS m/z: 360.16 [M + H]^+^. R_T_ = 13.218 min, purity = 95.60%. Melting point: 132–134 °C.

### 3.4. Ethyl 3-Benzyl-4-oxo-6-phenyl-4,5,6,7-tetrahydro-1H-indole-2-carboxylate (***9***)

NaNO_2_ (1.1 eq) and ethyl 3-oxo-4-phenylbutanoate (1.1 eq) were added to a solution of **9** (1.0 eq) in acetic acid (3 mL) at 0 °C. The reaction was allowed to come to room temperature and was stirred for 2 h; then zinc powder (2.2 eq) was added. The mixture was heated to 80 °C with vigorous stirring. After the reaction was finished by TLC, the mixture was poured into water and extracted with ethyl acetate. The organic layer was dried over Na_2_SO_4_, filtered, and concentrated. The residue was purified by HPLC to obtain compound **11** as a white solid (5.4 mg, yield 6%). ^1^H NMR (400 MHz, DMSO-*d*_6_) δ 12.13 (s, 1H, NH), 7.41–7.29 (m, 4H), 7.29–7.17 (m, 5H), 7.14–7.07 (m, 1H), 4.40 (s, 2H), 4.26 (q, *J* = 7.2 Hz, 2H, -OCH_2_-), 3.52–3.42 (m, 1H), 3.02 (d, *J* = 8.0 Hz, 2H), 2.87–2.77 (m, 1H), 2.49–2.41 (m, 1H), and 1.26 (t, *J* = 7.2 Hz, 3H, -CH_3_). R_T_ = 14.066 min, purity = 97.23%. Melting point: 135–137 °C.

### 3.5. N-(4-Methoxyphenyl)-1-methyl-1H-indole-3-carboxamide (***13***)

Following the general procedure A, compound **13** (65 mg, yield 58%) was obtained as a white solid. ^1^H NMR (400 MHz, DMSO-*d*_6_) δ 9.66 (s, 1H, NH), 8.33–8.15 (m, 2H), 7.70 (d, *J* = 8.8 Hz, 2H), 7.51 (d, *J* = 8.4 Hz, 1H), 7.29–7.16 (m, 2H), 6.92 (d, *J* = 8.8 Hz, 2H), 3.87 (s, 3H, -NCH_3_), and 3.74 (s, 3H, -OCH_3_). ^13^C NMR (101 MHz, DMSO-*d*_6_) δ 163.14, 155.37, 137.27, 133.38, 132.64, 127.27, 122.63, 121.78, 121.74, 121.31, 114.19, 110.78, 110.13, 55.60, and 33.50. LC-MS m/z: 281.13 [M + H]^+^. R_T_ = 8.755 min, purity = 97.08%. Melting point: 122–124 °C.

### 3.6. N-(4-Methoxyphenyl)benzo[D]thiazole-5-carboxamide (***15***)

Following the general procedure A, compound **15** (60 mg, yield 72%) was obtained as a white solid. ^1^H NMR (400 MHz, DMSO-*d*_6_) δ 10.37 (s, 1H, NH), 9.52 (s, 1H), 8.76 (d, *J* = 2.0 Hz, 1H), 8.30 (d, *J* = 8.4 Hz, 1H), 8.09 (dd, *J* = 8.4, 2.0 Hz, 1H), 7.76 (d, *J* = 8.8 Hz, 2H), 7.02–6.86 (m, 2H), and 3.75 (s, 3H, -OCH_3_). ^13^C NMR (101 MHz, DMSO-*d*_6_) δ 165.06, 158.20, 156.09, 153.32, 137.16, 133.69, 132.72, 125.19, 122.97, 122.56, 122.53, 114.21, and 55.63. LC-MS m/z: 285.07 [M + H]^+^. R_T_ = 8.727 min, purity = 96.02%. Melting point: 126–128 °C.

### 3.7. N-(4-Methoxyphenyl)benzo[D]thiazole-7-carboxamide (***16***)

Following the general procedure A, compound **16** (35 mg, yield 37%) was obtained as a yellowish solid. ^1^H NMR (400 MHz, DMSO-*d*_6_) δ 10.54 (d, *J* = 9.6 Hz, 1H, NH), 9.50 (d, *J* = 9.2 Hz, 1H), 8.48–8.21 (m, 2H), 7.83–7.68 (m, 3H), 6.98 (t, *J* = 9.2 Hz, 2H), and 4.03–3.38 (m, 3H, -OCH_3_). ^13^C NMR (101 MHz, DMSO-*d*_6_) δ 164.07, 160.38, 156.36, 154.66, 133.26, 132.16, 128.72, 126.93, 126.37, 124.60, 122.94, 114.24, and 55.62. R_T_ = 12.843 min, purity = 90.32%. Melting point: 120–122 °C.

### 3.8. N-(4-Methoxyphenyl)-3-(1H-pyrrol-1-Yl)benzamide (***17***)

Following the general procedure A, compound **17** (66 mg, yield 52%) was obtained as a white solid. ^1^H NMR (400 MHz, DMSO-*d*_6_) δ 10.26 (s, 1H, NH), 8.13 (s, 1H), 7.83 (dd, *J* = 7.6, 2.0 Hz 1H), 7.81–7.75 (m, 1H), 7.76–7.69 (m, 2H), 7.60 (t, *J* = 8.0 Hz, 1H), 7.48 (t, *J* = 2.4 Hz, 2H), 6.97 (d, *J* = 8.8 Hz, 2H), 6.33 (t, *J* = 2.4 Hz, 2H), and 3.76 (s, 3H, -OCH_3_). ^13^C NMR (101 MHz, DMSO-*d*_6_) δ 164.91, 156.17, 140.37, 137.00, 132.53, 130.28, 124.98, 122.60, 122.57, 119.66, 118.73, 114.25, 111.25, and 55.62. LC-MS m/z: 293.13 [M + H]^+^. R_T_ = 9.404 min, purity = 94.44%. Melting point: 118–120 °C.

### 3.9. N-(4-Methoxyphenyl)-3-(piperidin-1-Yl)benzamide (***18***)

Following the general procedure A, compound **18** (70 mg, yield 56%) was obtained as a white solid. ^1^H NMR (400 MHz, DMSO-*d*_6_) δ 10.05 (s, 1H, NH), 7.69 (d, *J* = 8.8 Hz, 2H), 7.46 (s, 1H), 7.38–7.27 (q, *J* = 7.6 Hz, 2H), 7.09 (d, *J* = 7.6 Hz, 1H), 6.93 (d, *J* = 8.4 Hz, 2H), 3.74 (s, 3H, -OCH_3_), 3.19 (t, *J* = 5.2 Hz, 4H), and 1.69–1.48 (m, 6H). ^13^C NMR (101 MHz, DMSO-*d*_6_) δ 166.02, 155.96, 151.99, 136.26, 132.79, 129.35, 122.53, 118.98, 117.97, 115.08, 114.12, 55.60, 49.91, 25.67, 25.59, and 24.34. LC-MS m/z: 311.17 [M + H]^+^. R_T_ = 13.214 min, purity = 95.79%. Melting point: 122–124 °C.

### 3.10. MAT2A Enzymatic Assay

The inhibition of MAT2A by each compound was determined using the colorimetric Phosphate Assay Kit-PiColorLock (#ab270004) as described previously [18]. Briefly, serially dilute the stock solution of 10 mM 3-fold in DMSO to obtain a nine-point dilution curve with final compound concentrations ranging from 100 μM to 15.2 nM. Assay measurements were performed in 1X buffer containing 50 mM Tris, 50 mM KCl, 10 mM MgCl_2_, and 0.05% Brij-35 (pH 8.0) in a final assay volume of 5 μL. A total of 2.5 μL of MAT2A protein with a final concentration of 20 μg/mL was added to each well of a 384 well assay plate containing 100 nL of each concentration of test compound dissolved in DMSO and incubated for 30 min at room temperature. ATP (100 μM final concentration) and L-Met (100 μM final concentration) were subsequently added and incubated for 30 min at room temperature. Then 20 μL of the phosphate assay kit substrate was added to each well, incubated at room temperature for 30 min, and then the absorbance was measured at 620 nm on a plate reader.

### 3.11. Cellular Assay

Cell viability assays were performed using Cell Counting Kit-8 (CCK8). Briefly, the MTAP-deleted HCT116 cells (Horizon) were plated at 1000 cells per well in 96-well plates in RPMI-1640 medium with 10% FBS and 1% PS, and then incubated at 37 °C under a 5% CO_2_ atmosphere overnight. The stock solutions were serially diluted 3-fold in DMSO to obtain an eight-point dilution curve with final compound concentrations and test compounds added. After 72 h, 10 μL CCK-8 dye was added in each well and the cells were incubated for 2–4 h. Fluorescence was measured at 450 nm. Dose response curves were analyzed in GraphPad Prism 8.

### 3.12. Virtual Screening

The pharmacophore modeling, virtual screening, and docking studies were carried out using Schrödinger Small-Molecule Drug Discovery Suite (Small-Molecule Drug Discovery Suite 2019-1, Schrödinger, LLC, New York, NY, USA, 2019). The X-ray crystal structure of the MAT2A (PDB ID: 7KCC) was retrieved from the Protein Data Bank. The protein structure was processed by *protein preparation wizard*. The hydrogen bonds assignment tool was implemented for optimizing the network of H-bonds. The minimization of energy was carried out by an OPSL3 force field. The generation of a grid around the allosteric binding site by selecting a co-crystalized molecule was performed by a receptor grid generation panel of *Glide*. The grid size was kept default at 20 Ǻ. Compounds of ChemDiv chemical library were downloaded and prepared by *Ligprep* for geometric minimization using the OPLS force field. Virtual screening was carried out in docking approach by high throughput virtual screening (HTVS), standard precision (SP), and extra precision (XP). HTVS, SP, and XP differ only in number penalties. A total of 5% of the best scoring compounds from HTVS were again assessed in SP docking for high accuracy. The best 5% of compounds from SP docking were further evaluated in XP docking mode. The best 5% of compounds from XP docking were further evaluated in a pharmacophore model and with visual inspection.

### 3.13. Reagents and Compounds

Compounds used for MAT2A inhibitor screening (see Table S3) were purchased from the ChemDiv (Chemdiv, San Diego CA, USA) (https://www.chemdiv.com (1 September 2024)) with a purity of more than 95%. **AZ-28** and **AG-270** were purchased from Bidepharm Co., Ltd. (Shanghai, China).

### 3.14. In Vivo Tumor Xenograft Model

The antitumor efficacy of compound **17** was evaluated in female BALB/c nude mice using a standardized tumor xenotransplantation model. Animals were maintained under specific-pathogen-free (SPF) conditions, and all experimental procedures adhered to institutional animal care protocols approved by the Institute of Radiation Medicine, the Chinese Academy of Medical Sciences, and Peking Union Medical College, complying with national guidelines for humane treatment. For tumor induction, 2 × 10^6^ HCT-116 MTAP^−/−^ cells suspended in PBS were subcutaneously implanted into the right hind flank. Following a 7-day engraftment period, mice were stratified into three cohorts (n = 5/group) via block randomization performed by an independent researcher to minimize selection bias. Compound **17** was administered orally at 30 mg/kg once daily, using 5% DMSO + 10% Solutol HS-15 + 85% saline as the vehicle solution. The positive control AG-270 was dosed at the same regimen (30 mg/kg, orally, once daily) with identical vehicle composition. Tumor progression was monitored every other day through digital caliper measurements, with volumes calculated using the ellipsoid approximation formula: V = 0.5 × length × width^2^. The antitumor effect of the compound was assessed by tumor growth inhibition (TGI) or relative tumor proliferation rate (T/C): TGI (%) = [1 − (V_t1_ − V_t0_)/(V_c1_ − V_c0_)] × 100%, where V_c1_ and V_t1_ are the mean volumes of control and treated groups at the time of tumor extraction, while V_c0_ and V_t0_ are the same groups at the start of dosages; T/C (%) = TRTV/CRTV × 100%, where TRTV is the relative tumor volume (RTV) of treated groups, while CRTV is the RTV of control groups (RTV = V_t_/V_0_, V_t_ is the mean volume of treated groups at the time of tumor extraction, and V_0_ is the mean volume of the same groups at the start of dosages).

## 4. Conclusions

In this study, a series of novel MAT2A inhibitors were identified through a multistep virtual screening strategy targeting the allosteric binding site, followed by systematic scaffold optimization and structure–activity relationship (SAR) exploration. Initial hits **A10** (IC_50_ = 4.6 μM) and **A11** (IC_50_ = 2.5 μM), selected from the ChemDiv library via pharmacophore modeling and molecular docking, revealed critical interactions such as hydrogen bonding between the methoxyphenyl moiety and Gly-193, as well as hydrophobic embedding of the pyrrole ring within the Phe-20/Trp-274 cavity. Scaffold-hopping strategies demonstrated that conformational flexibility significantly enhanced activity, with the open-chain derivative **A11** outperforming rigid cyclic analogs (e.g., **8**, IC_50_ = 6.8 μM). Further optimization highlighted the importance of hydrophobic and π-π interactions, exemplified by compound **15** (IC_50_ = 0.91 μM), where a biaryl group improved potency 2.7-fold via face-to-face π-π stacking with Phe-18. Conversely, steric hindrance from bulky substituents (e.g., benzo[1,3]dioxole in **14**) or unfavorable conformations (e.g., perpendicular bicyclic systems in **16**) abolished activity entirely, underscoring the sensitivity of the binding pocket to steric clashes. Hydrogen bond stability emerged as a key determinant of potency, as the removal of the methoxy group in derivatives like **9** and **10** disrupted interactions with Gly-193, rendering them inactive. The optimized compound **17** (IC_50_ = 0.43 μM), featuring a biaryl scaffold and amide linker, exhibited robust enzymatic and cellular activity (IC_50_ = 1.4 μM in MTAP^−/−^ HCT116 cells), supported by molecular docking showing hydrogen bonds with Arg-313 and π-π interactions with Phe-18. In vivo, **17** achieved 58.4% tumor growth inhibition at 30 mg/kg (q.d.) without adverse effects on body weight or survival, demonstrating both efficacy and tolerability. These results elucidate the synergistic roles of hydrogen bonding, hydrophobic packing, and conformational adaptability in MAT2A inhibition, providing a structural foundation for designing selective therapeutics against MTAP-deleted cancers.

## Figures and Tables

**Table 1:** MAT2A Inhibitory Activities of Compounds **A10, 9**−**11**.

Compound No	Structure	IC_50_ (μM) ^a^	Maximum Inhibition (%)
**A10**	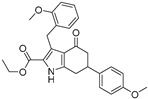	4.6 ± 0.3	65
**8**	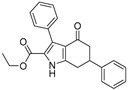	6.8 ± 0.2	47
**9**	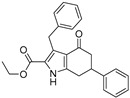	NA ^b^	/
**10**	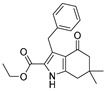	NA	/

^a^ The data were shown as mean ± SD from at least three independent experiments. ^b^ NA: no activity at 10 μM.

**Table 2:** MAT2A Inhibitory Activities of Compounds **12**–**19**.

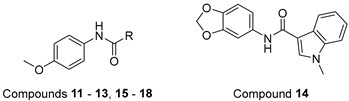			
Compound No	R	IC_50_ (μM) ^a^	Maximum Inhibition (%)
**A11**	/	2.5 ± 0.3	76
**11**	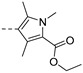	15.6 ± 3.1	/
**12**	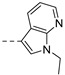	2.9 ± 0.2	86
**13**	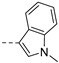	2.1 ± 0.6	82
**14**	/	NA ^b^	/
**15**	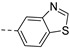	0.91 ± 0.09	80
**16**	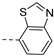	NA ^b^	/
**17**	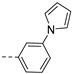	0.43 ± 0.06	84
**18**	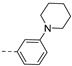	0.78 ± 0.15	72
**6 (AZ-28)**	/	0.11 ± 0.02	96

^a^ The data were shown as mean ± SD from at least three independent experiments. ^b^ NA: no activity at 10 μM.

**Table 3:** Antiproliferative Activity of Selected Compounds ^a^.

Compound No	MTAP^−/−^ HCT116 IC_50_ (μM)	Parental HCT116 IC_50_ (μM)
**A11**	6.0 ± 0.4	>10
**15**	2.2 ± 0.1	>10
**17**	1.4 ± 0.3	>10
**6 (AZ-28)**	0.35 ± 0.13	1.9 ± 0.2

^a^ The data were shown as mean ± SD from at least three independent experiments.

## Data Availability

The original contributions presented in this study are included in the article. Further inquiries can be directed to the corresponding authors.

## Supplementary Materials

The following supporting information can be downloaded at: https://www.mdpi.com/article/10.3390/molecules30102134/s1, Figure S1. The pharmacophore models generated; Figure S2. The ROC curves of model 6, model 9 and model 10; Figure S3. Structure and MAT2A inhibitory activity of compounds **A1–A11**; Figure S4. Overlay of compounds **A10** and **A11** at allosteric site; Figure S5. Surface models of the binding modes of compounds **A10** and **A11**; Table S1. The reported MAT2A allosteric inhibitors; Table S2. The enrichment score of each pharmacophore model; Table S3. The information for compounds **A1–A11**, **11**, **12** and **14**; Table S4. In vivo tumor xenograft model profiles of compound **17**; Table S5. The cytotoxicity of new compounds

## Abbreviations

The following abbreviations are used in this manuscript:

EF	Enrichment factor
MAT2A	Methionine adenosyltransferase 2A
MTAP	Methylthioadenosine phosphorylase
PRMT5	Protein arginine methyltransferase 5
ROC	Receiver operating characteristic
SAM	S-adenosylmethionine
SAR	Structure–activity relationship
SP	Standard precision
XP	Extra precision
2D	Two-dimensional
