# Supplementary material for: Discovery of Potential Scaffolds for Methionine Adenosyltransferase 2A (MAT2A) Inhibitors: Virtual Screening, Synthesis, and Biological Evaluation
Source: Molecules. 2025 May 12;30(10):2134. doi: 10.3390/molecules30102134 (PMC12113668; doi:10.3390/molecules30102134)
Supplement: Supplementary file 1 [file molecules-30-02134-s001.zip › molecules-3589334-supplementary.pdf]

Discovery of potential scaffolds for methionine adenosyltransferase 2A (MAT2A) inhibitors: virtual screening, synthesis, and biological evaluation

1. **Figure S1.** The pharmacophore models generated.
2. **Figure S2**. The ROC curves of model 6, model 9 and model 10.
3. **Figure S3.** Structure and MAT2A inhibitory activity of compounds **A1** – **A11**.
4. **Figure S4.** Overlay of compounds **A10** and **A11** at allosteric site.
5. **Figure S5.** Surface models of the binding modes of compounds **A10** and **A11.**
6. **Table S1** The reported MAT2A allosteric inhibitors.
7. **Table S2** The enrichment score of each pharmacophore model.
8. **Table S3** The information for compounds **A1 – A11, 11, 12 and 14**.
9. **Table S4**. *In vivo* tumor xenograft model profiles of compound **17**.
10. **Table S5**. The cytotoxicity of new compounds
11. ^{1}H NMR and ^{13}C NMR of new compounds
12. MS spectrum of new compounds
13. HPLC purity of new compounds

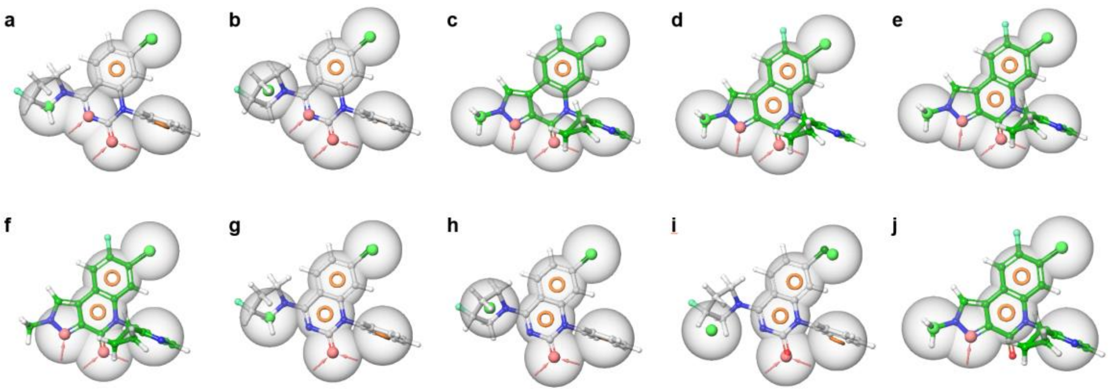

Figure S1. The pharmacophore models generated. The pharmacophore features are represented as follows: hydrogen-bond acceptor (A; red sphere with vectors), hydrogen-bond donor (D; blue sphere with vector), aromatic group (R; orange ring),. The shared pharmacophore features were shown as the superposition of the compound with the highest fitness score. a) Model 1 (AAHHRR_1); b) Model 2 (AAHHRR_2); c) Model 3 (AAHHRR_3); d) Model 4 (AAHHRR_4); e) Model 5 (AAHHRRR_1); f) Model 6 (AAHRRR_1); g) Model 7 (AHHRRR_1); h) Model 8 (AHHRRR_2); i) Model 9 (AHHRRR_3); j) Model 10 (AHHRRR_4).

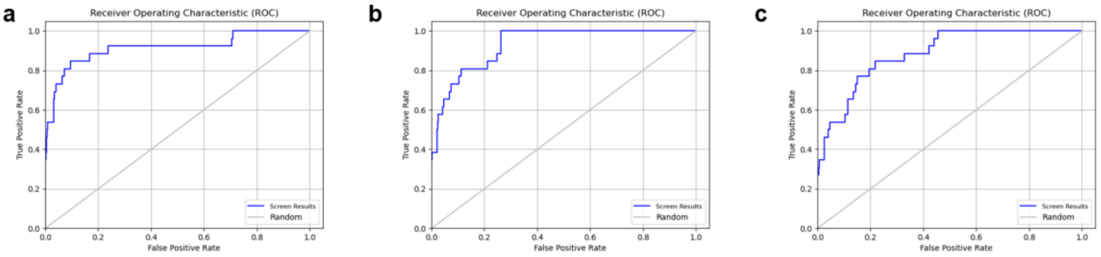

Figure S2. The ROC curves of model 6, model 9 and model 10. a) ROC curve of model 6 (AAHRRR_1), ROC = 0.91; b) ROC curve of model 9 (AHHRRR_3), ROC = 0.93; c) ROC curve of model 10 (AHHRRR_4), ROC = 0.88.

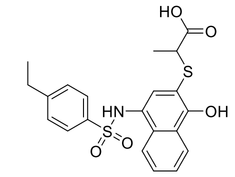

A1 (8018-7349)
MAT2A inhibition: 11 ± 3 %

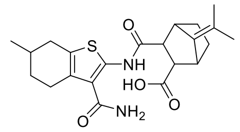

A2 (Y204-5799)
MAT2A inhibition: no inhibition

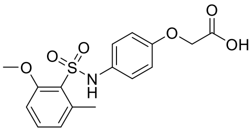

A3 (8013-3831)
MAT2A inhibition: no inhibition

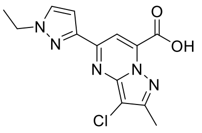

A4 (Y504-9451)
MAT2A inhibition: 36 ± 5 %

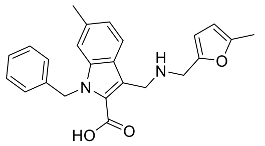

A5 (E518-1567)
MAT2A inhibition: 22 ± 5 %

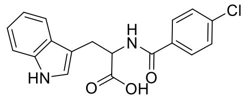

A6 (1682-7513)
MAT2A inhibition: no inhibition

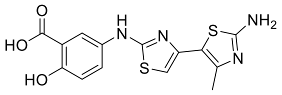

A7 (7100-1514)
MAT2A inhibition: no inhibition

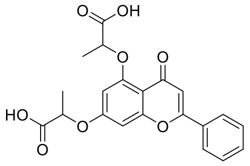

A8 (Y041-2308)
MAT2A inhibition: no inhibition

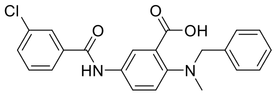

A9 (G889-0186)
MAT2A inhibition: 35 ± 2 %

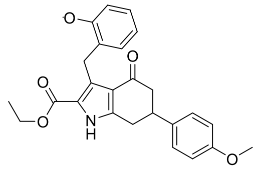

A10 (C276-1403)
MAT2A inhibition: 65 ± 5 %

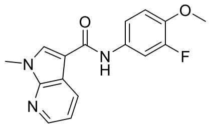

A11 (P168-0361)
MAT2A inhibition: 70 ± 2 %

Figure S3. Structure and MAT2A inhibitory activity of compounds **A1** – **A11**. The inhibition of MAT2A of each compound was measured at 10 μM. Values are the mean of three experiments.

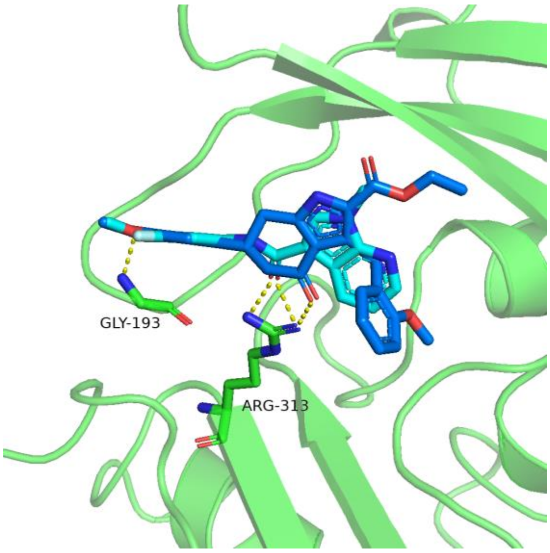

Figure S4. Overlay of compounds **A10** and **A11** at allosteric site.

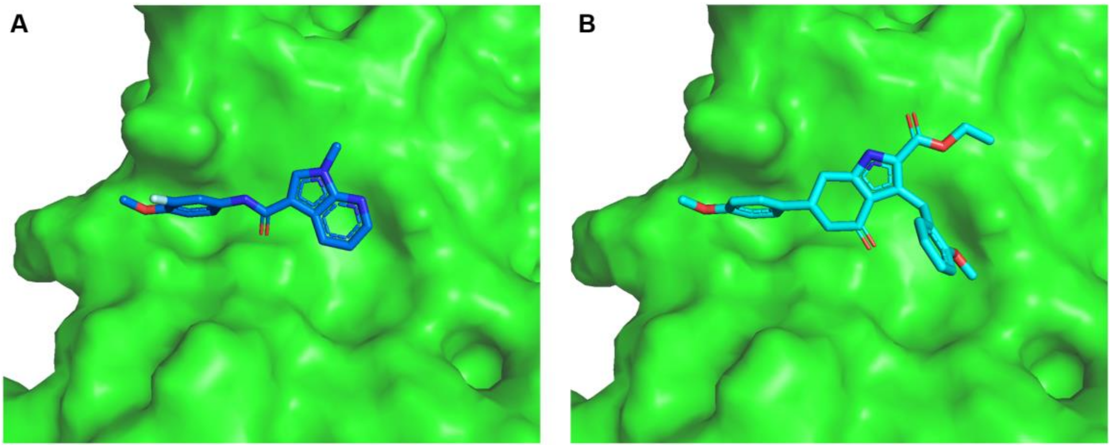

Figure S5. Surface models of the binding modes of compounds **A10** (left) and **A11** (right) at the allosteric binding site.

Table S1 The reported MAT2A allosteric inhibitors.

Compound	Structure	PDB id	Provenance
PF-9366	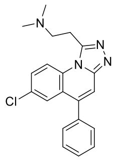	5UGH	*Nat Chem Biol.* **2017**; 13(7):785-792.
Cmpd **28**	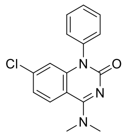	7BHV	*J Med Chem.* **2021**, 64, 6814-6826
Cmpd **31**	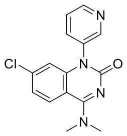	7BHX	
Cmpd **32**	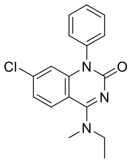	/	
Cmpd **33**	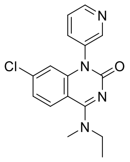	/	
AG-270	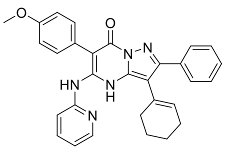	7KCC	*J Med Chem.* **2021**, 64, 4430-4449
AG-24512	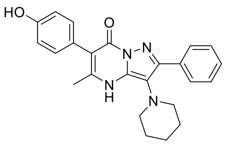	7KCF	

Cmpd **21**	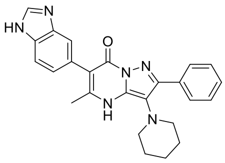	/	
Cmpd **23**	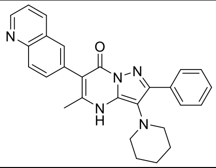	/	
Cmpd **35**	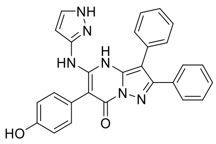	7KDB	
AGI-43192	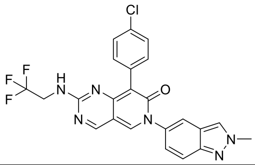	7RWG	*J. Med. Chem.* **2022**, 65, 4600−4615
AGI-41998	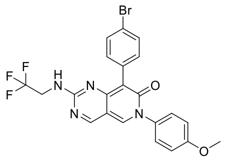	7RWH	
Cmpd **14**	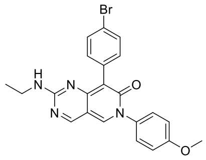	/	
Cmpd **21**	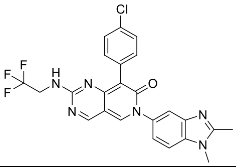	/	
IDEAYA cmpd A	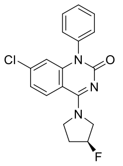	8P4H	*Bioorg Med Chem Lett.* **2023**; 94:129450
Cmpd **4**	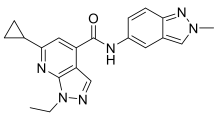	/	
Cmpd **9**	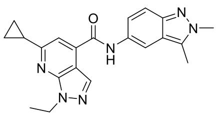	/	
Cmpd **17**	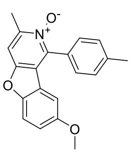	/	

Cmpd **14**	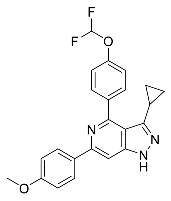	/	
Cmpd **15**	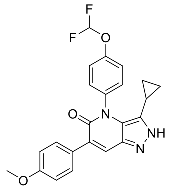	8QE1	
Cmpd **21**	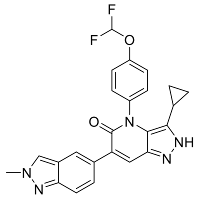	8QE2	*J Med Chem.* **2024**, 67, 4541-4559
Cmpd **31**	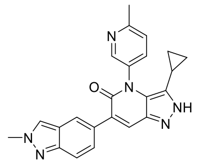	8QE3	
Cmpd **37**	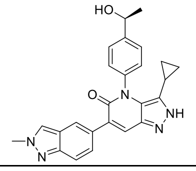	/	
Cmpd **40**	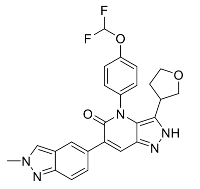	/	
Cmpd **21**	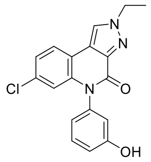	/	*J Med Chem.* **2024**, 67, 9431-9446
Cmpd **39**	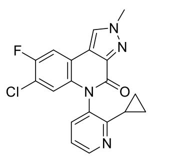	8XB0	

Table S2 The enrichment score of each pharmacophore model.

Hypothesis	Feature	EF1%	ROC
Model 1	AAHHRR_1	30.83	0.70
Model 2	AAHHRR_2	30.83	0.70
Model 3	AAHHRR_3	30.83	0.88
Model 4	AAHHRR_4	26.98	0.75
Model 5	AAHHRRR_1	30.83	0.87

Model 6	AAHRRR_1	34.68	0.91
Model 7	AHHRRR_1	34.68	0.87
Model 8	AHHRRR_2	34.68	0.87
Model 9	AHHRRR_3	34.68	0.93
Model 10	AHHRRR_4	26.98	0.88

Table S3 The information for compounds **A1 – A11, 11, 12 and 14**.

Compound No.	ChemDiv ID	Smiles
**A1**	8018-7349	CCc1ccc(cc1)S(Nc1cc(c(c2ccccc12)O)SC(C)C(O)=O)(=O)=O
**A2**	Y204-5799	CC1CCc2c(C(N)=O)c(NC(C3C4CCC(C3C(O)=O)C4=C(C)C)=O)sc2C1
**A3**	8013-3831	Cc1ccc(cc1S(Nc1ccc(cc1)OCC(O)=O)(=O)=O)OC
**A4**	Y504-9451	CCn1ccc(c2cc(C(O)=O)n3c(c(c(C)n3)[Cl])n2)n1
**A5**	E518-1567	Cc1ccc2c(CNCc3ccc(C)o3)c(C(O)=O)n(Cc3ccccc3)c2c1
**A6**	1682-7513	C(C(C(O)=O)NC(c1ccc(cc1)[Cl])=O)c1c[nH]c2ccccc12
**A7**	7100-1514	Cc1c(c2csc(Nc3ccc(c(c3)C(O)=O)O)n2)sc(N)n1
**A8**	Y041-2308	CC(C(O)=O)Oc1cc(c2C(C=C(c3ccccc3)Oc2c1)=O)OC(C)C(O)=O
**A9**	G889-0186	CN(Cc1ccccc1)c1ccc(cc1C(O)=O)NC(c1cccc(c1)[Cl])=O
**A10**	C276-1403	CCOC(c1c(Cc2ccccc2OC)c2C(CC(Cc2[nH]1)c1ccc(cc1)OC)=O)=O
**A11**	P168-0361	Cn1cc(C(Nc2ccc(c(c2)F)OC)=O)c2cccnc12
**10**	C276-0628	CCOC(c1c(Cc2ccccc2)c2C(CC(C)(C)Cc2[nH]1)=O)=O
**11**	C722-0383	CCOC(c1c(C)c(C(Nc2ccc(cc2)OC)=O)c(C)n1C)=O
**12**	P168-0459	CCn1cc(C(Nc2ccc(cc2)OC)=O)c2cccnc12
**14**	Y044-3661	Cn1cc(C(Nc2ccc3c(c2)OCO3)=O)c2ccccc12

Table S4. *In vivo* tumor xenograft model profiles of compound **17**.

Compound	Tumor volume (mm^3)[a]	T/C (%)[b]	TGI (%)[b]
**Vehicle**	1517.7 ± 350.2		
**17**	659.9 ± 100.2	44.4	58.4
**AG-270**	351.9 ± 96.1	22.9	80.4

[a] Mean values ± SD

[b] The inhibition of tumor growth was calculated by T/C and TGI

Table S5. The cytotoxicity of new compounds

Compound	HEK293A IC_{50} (μM)
**A10**	55.6 ± 7.8
**A11**	>100
**8**	>100
**9**	>100
**10**	>100
**11**	>100
**12**	>100
**13**	>100
**14**	>100
**15**	>100
**16**	>100
**17**	>100
**18**	>100

^{1}H NMR of compound **8** (400 MHz, DMSO-d_6)

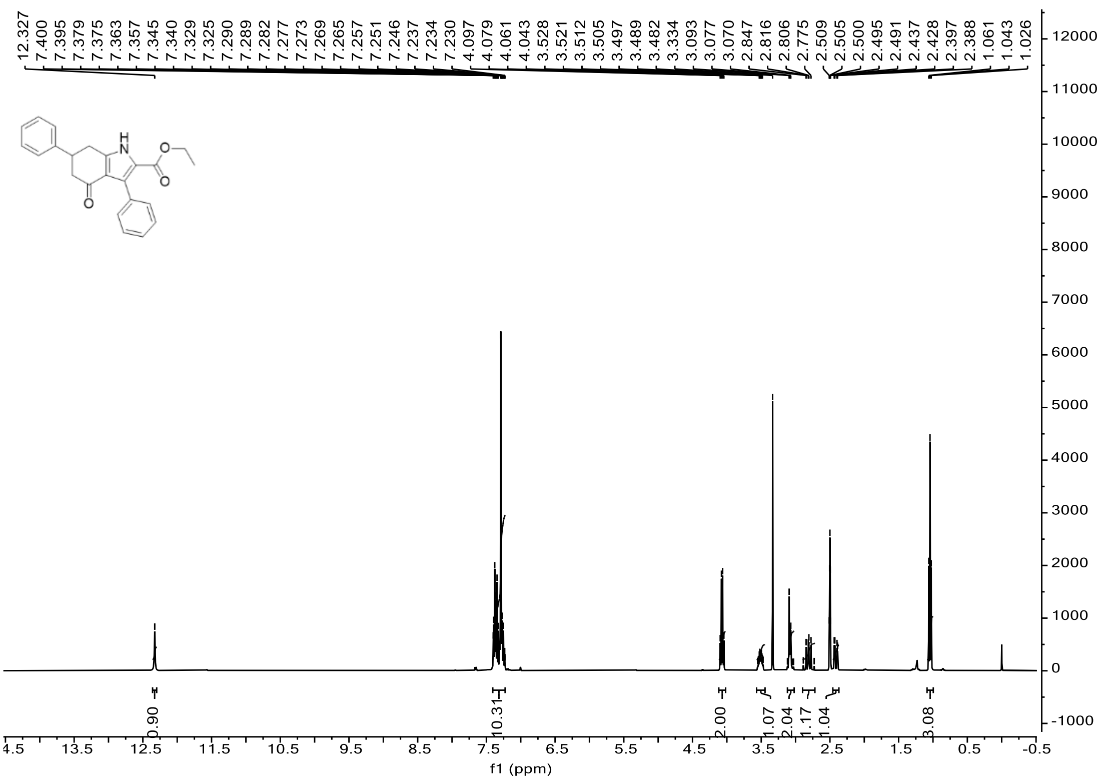

^{13}C NMR of compound **8** (100 MHz, DMSO-d_6)

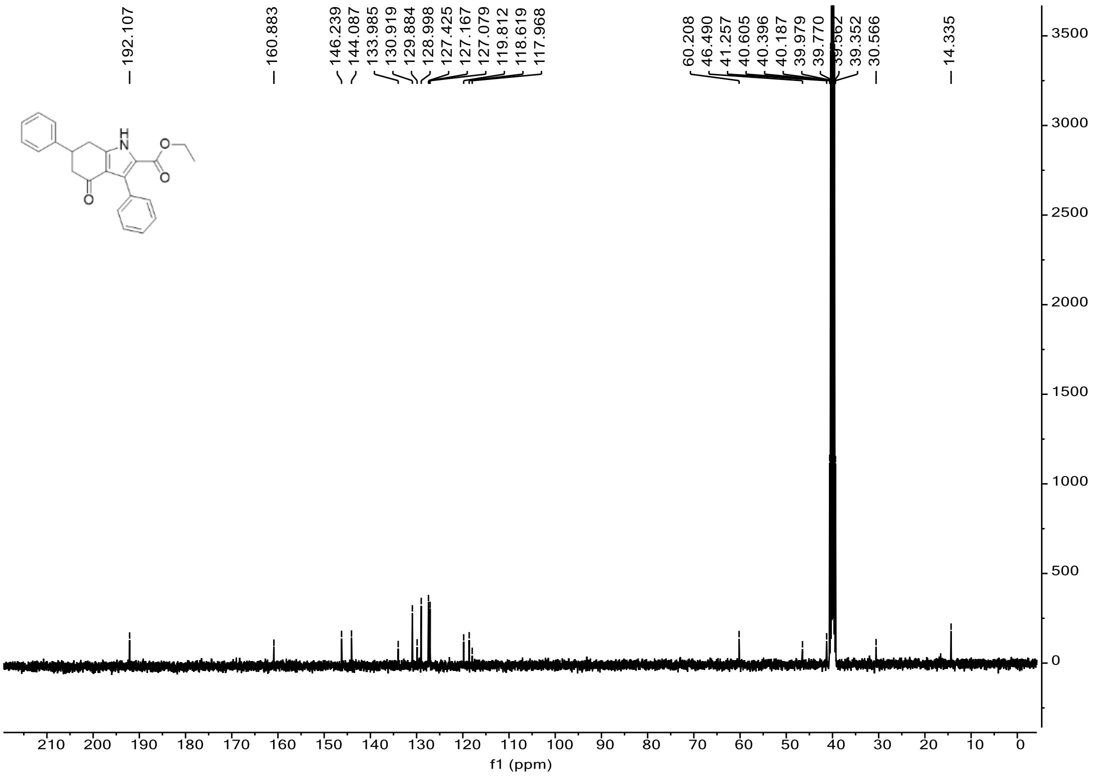

^{1}H NMR of compound **9** (400 MHz, DMSO-d_6)

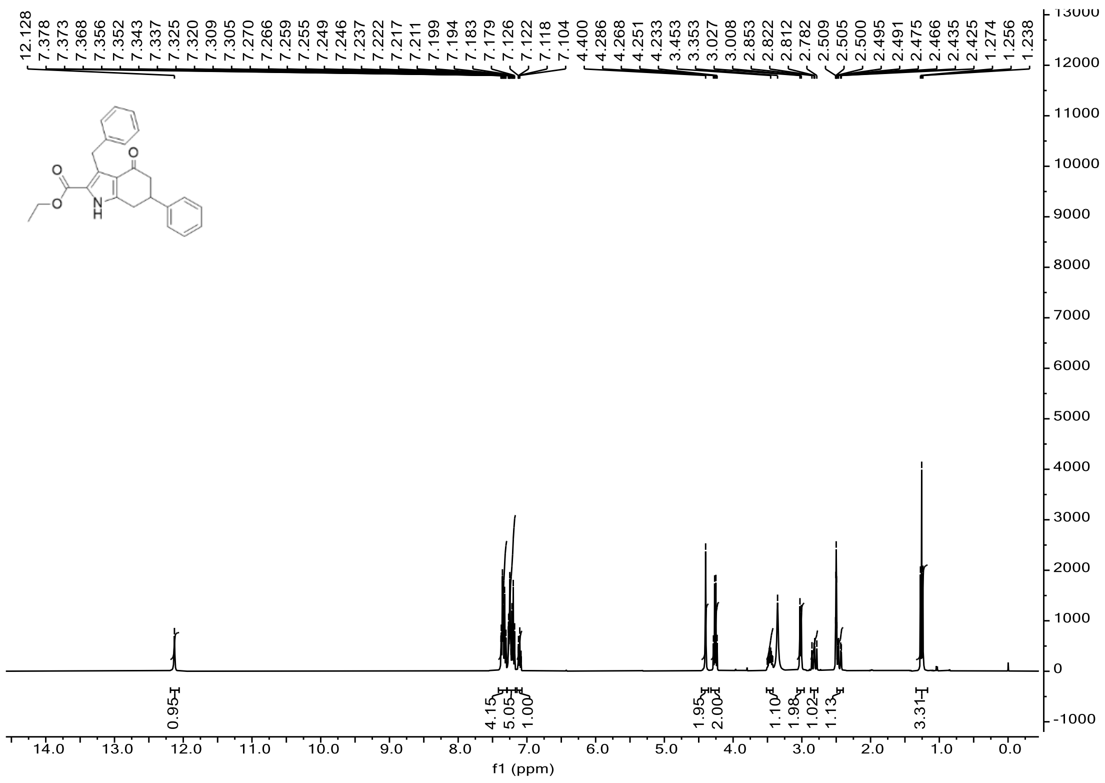

^{1}H NMR of compound **13** (400 MHz, DMSO-d_6)

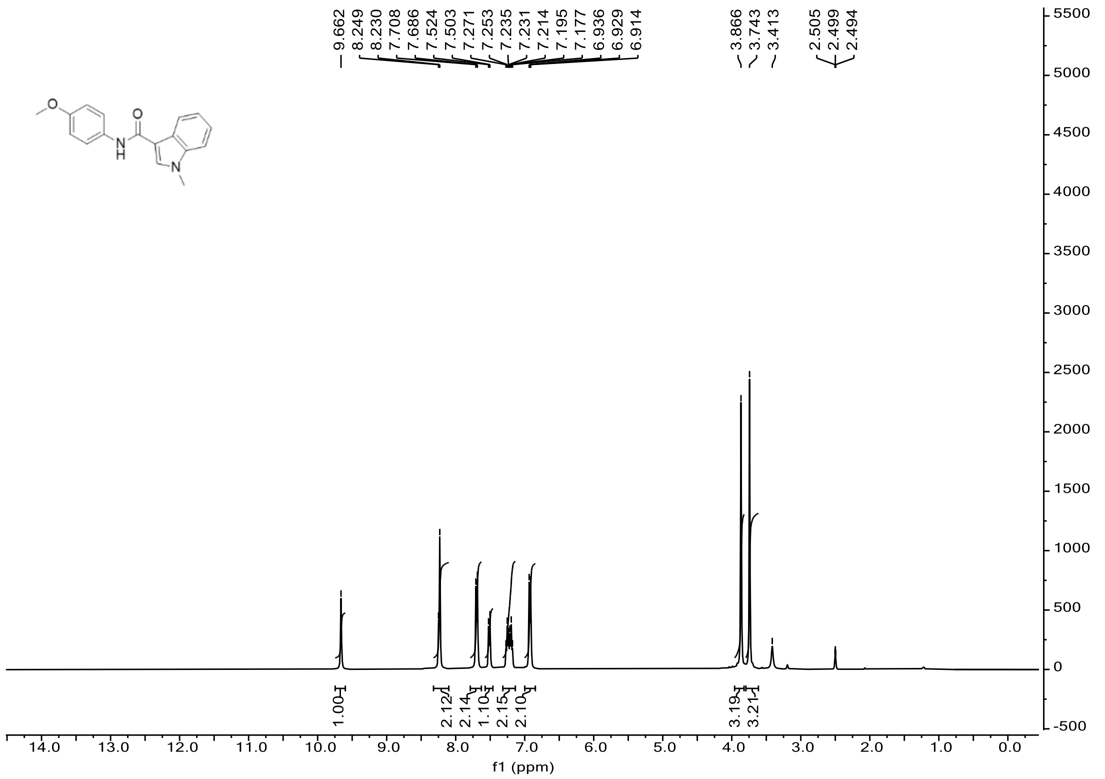

^{13}C NMR of compound **13** (100 MHz, DMSO-d_6)

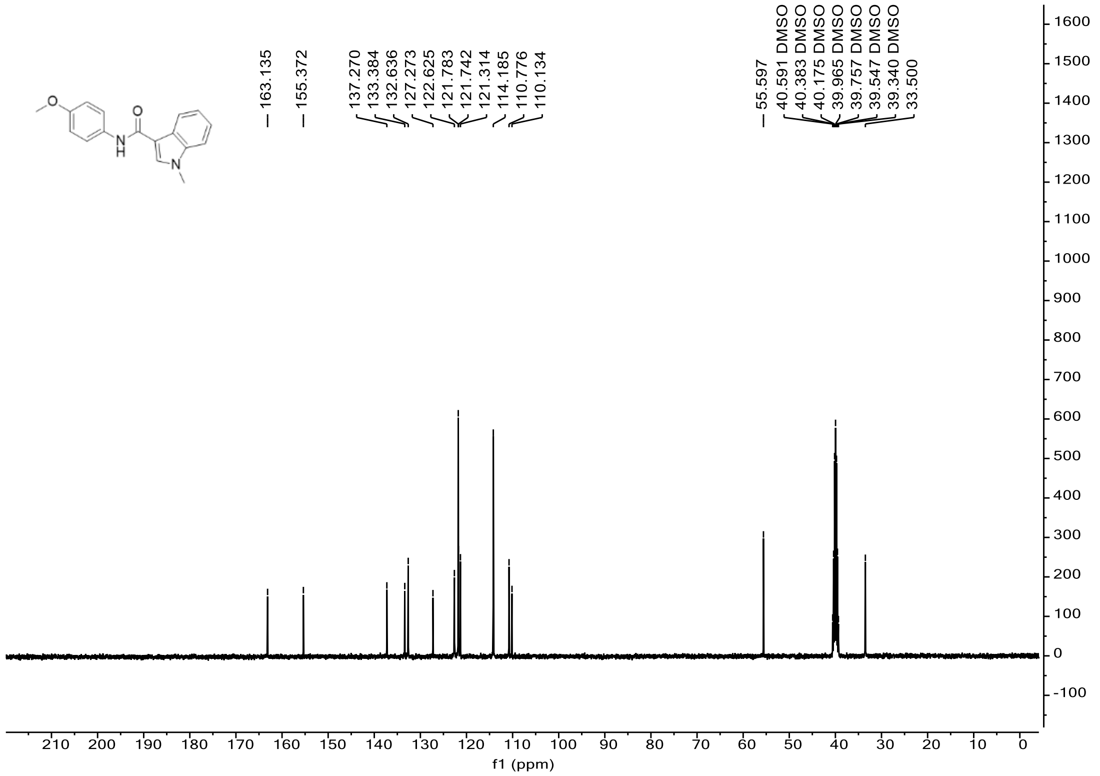

^{1}H NMR of compound **15** (400 MHz, DMSO-d_6)

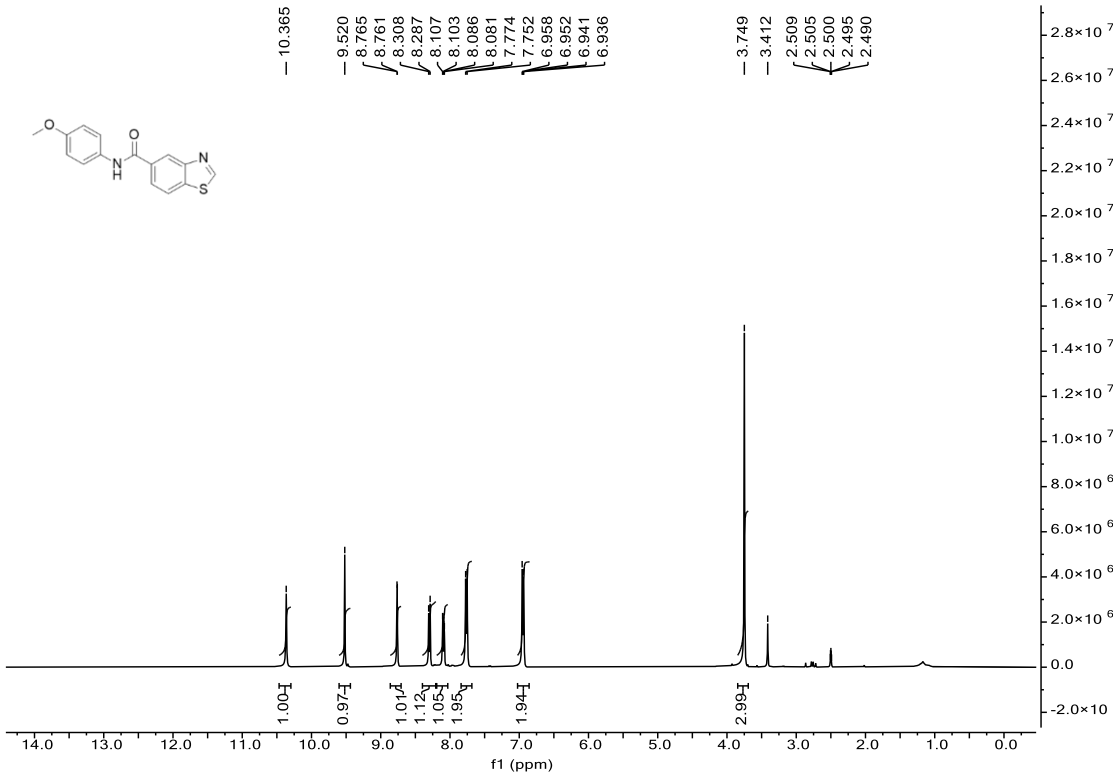

^{13}C NMR of compound **15** (100 MHz, DMSO-d_6)

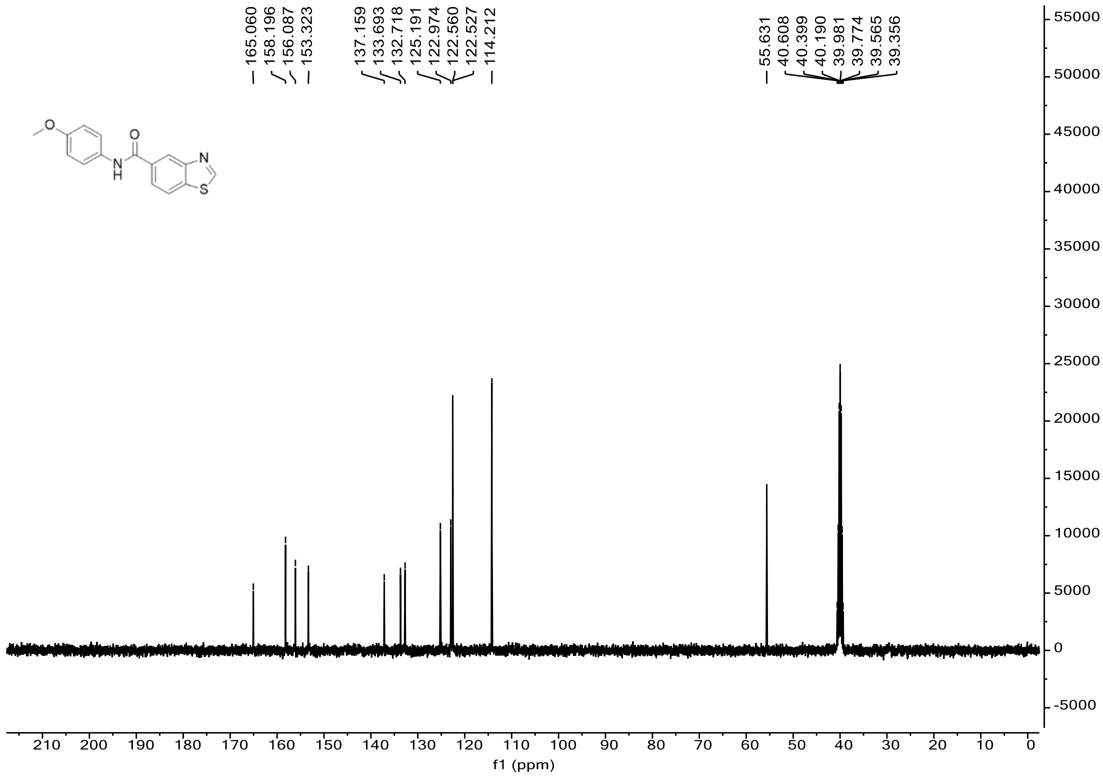

^{1}H NMR of compound **16** (400 MHz, DMSO-d_6)

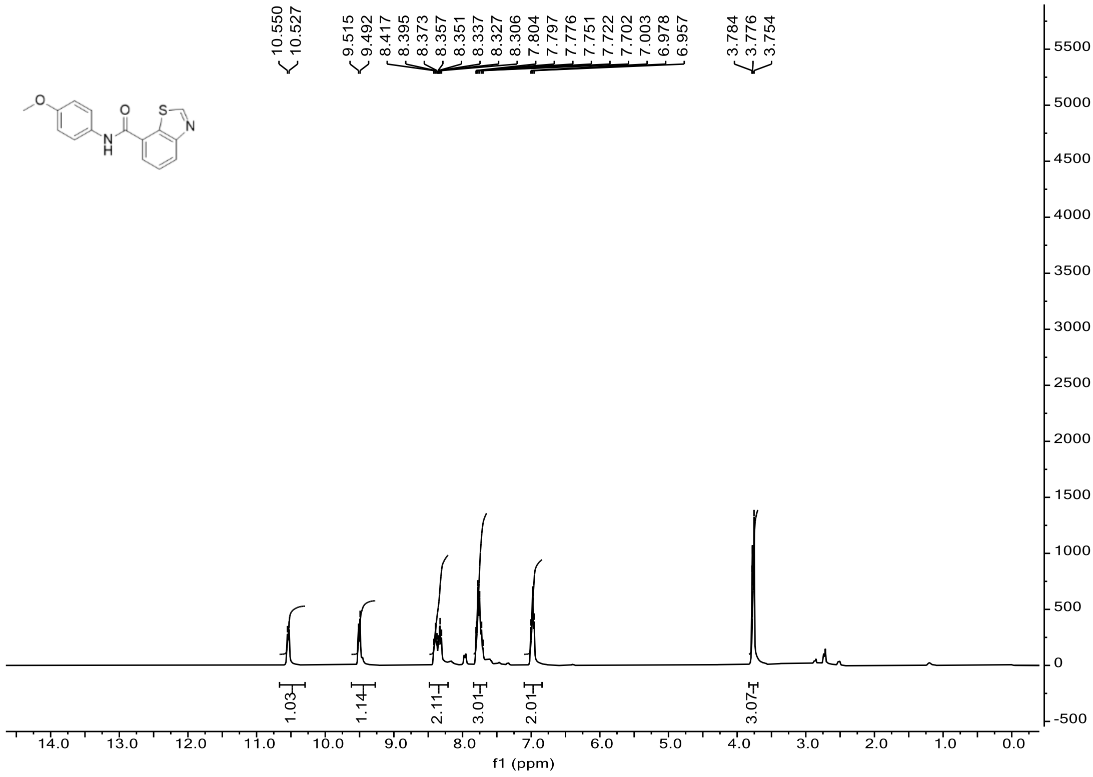

^{13}C NMR of compound **16** (100 MHz, DMSO-d_6)

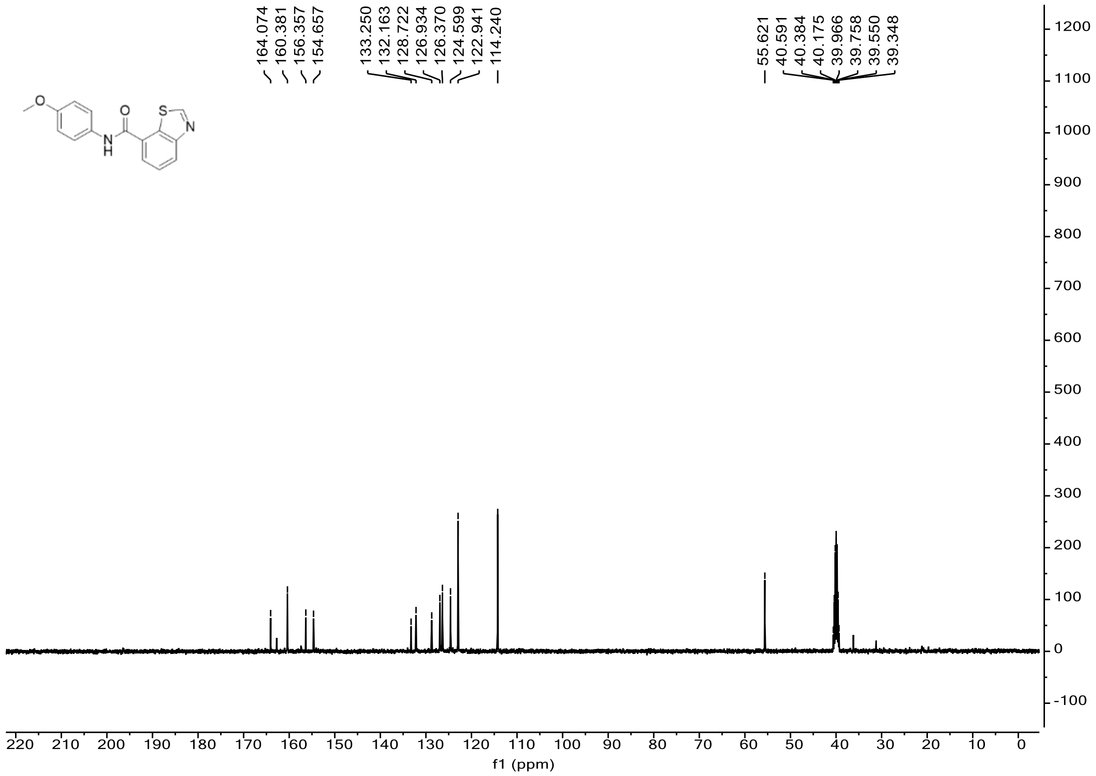

^{1}H NMR of compound **17** (400 MHz, DMSO-d_6)

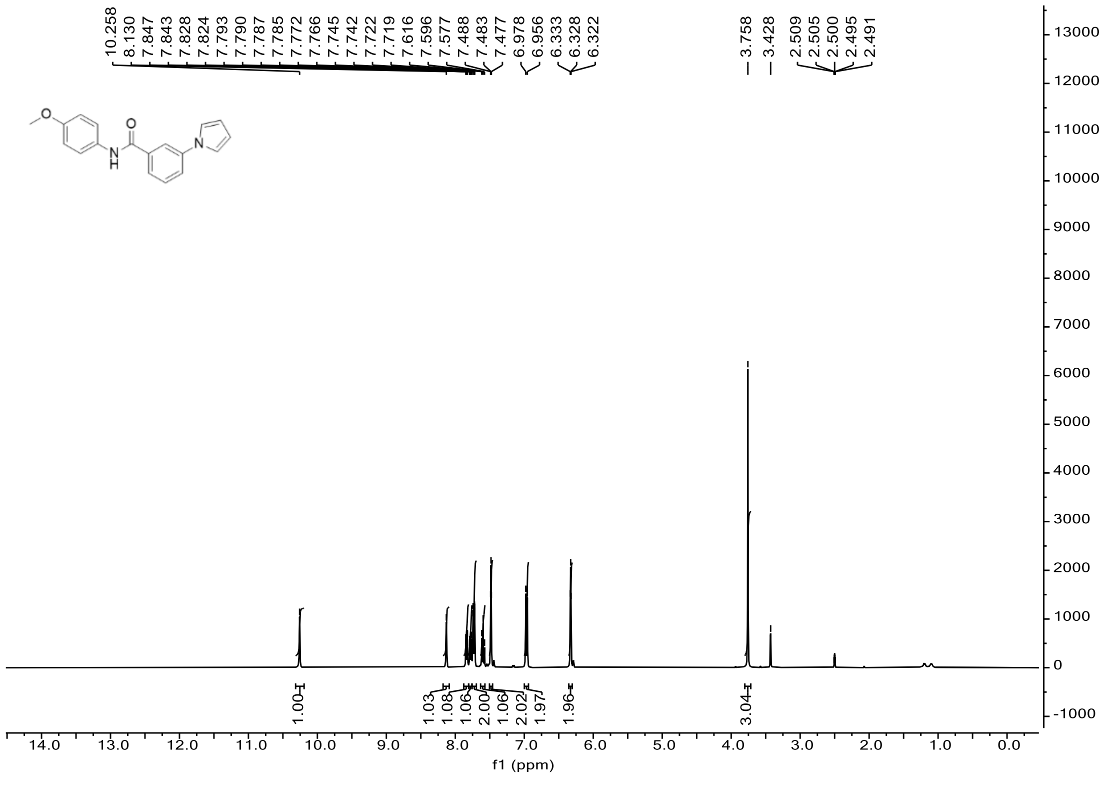

^{13}C NMR of compound **17** (100 MHz, DMSO-d_6)

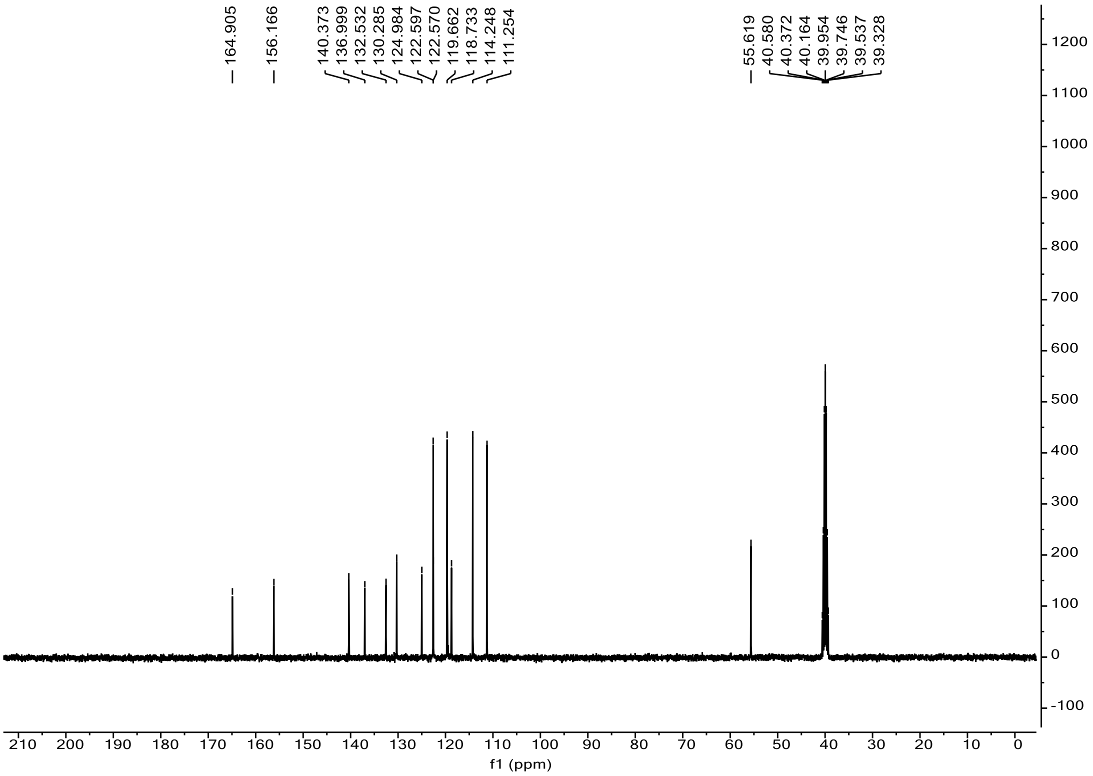

^{1}H NMR of compound **18** (400 MHz, DMSO-d_6)

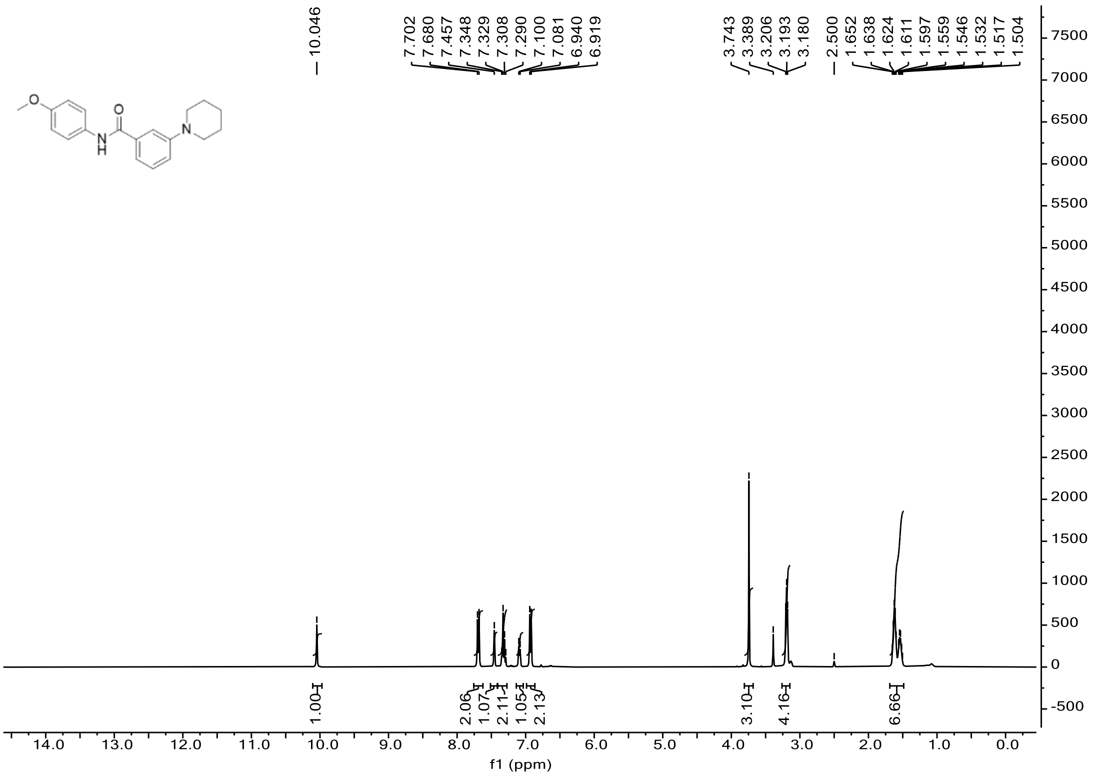

^{13}C NMR of compound **18** (100 MHz, DMSO-d_6)

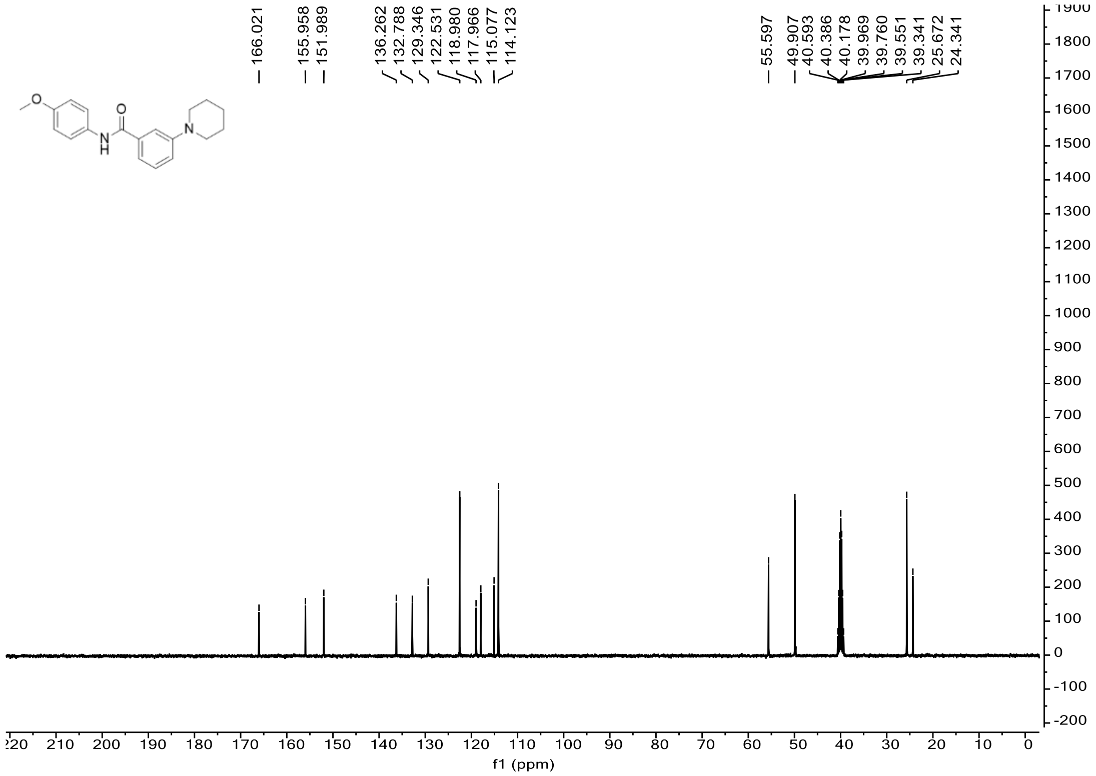

MS spectrum of compound **A10**

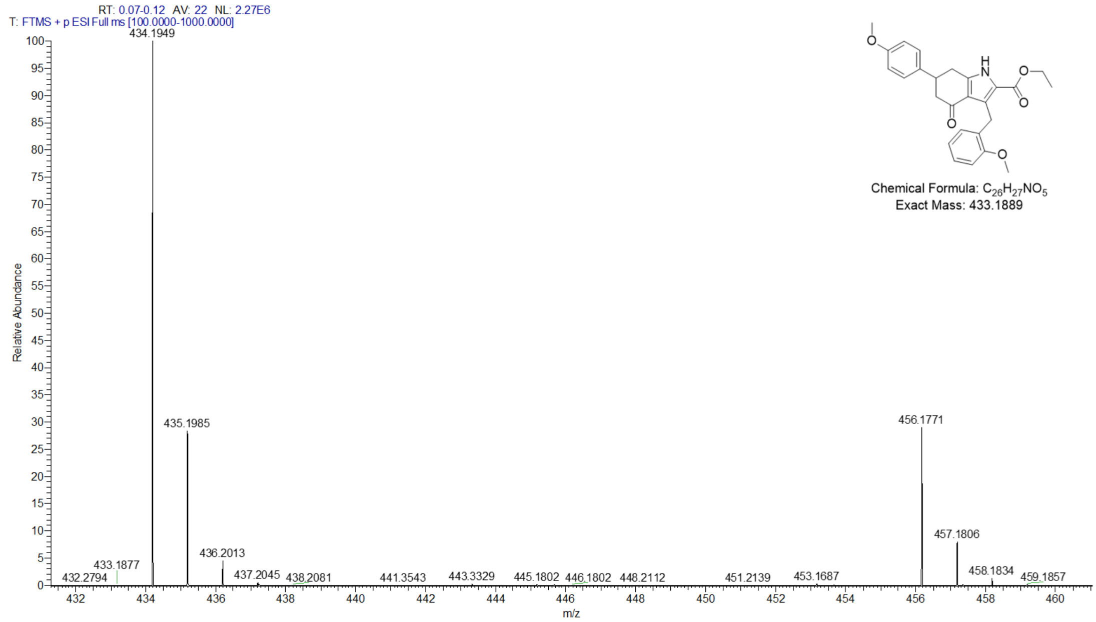

MS spectrum of compound **8**

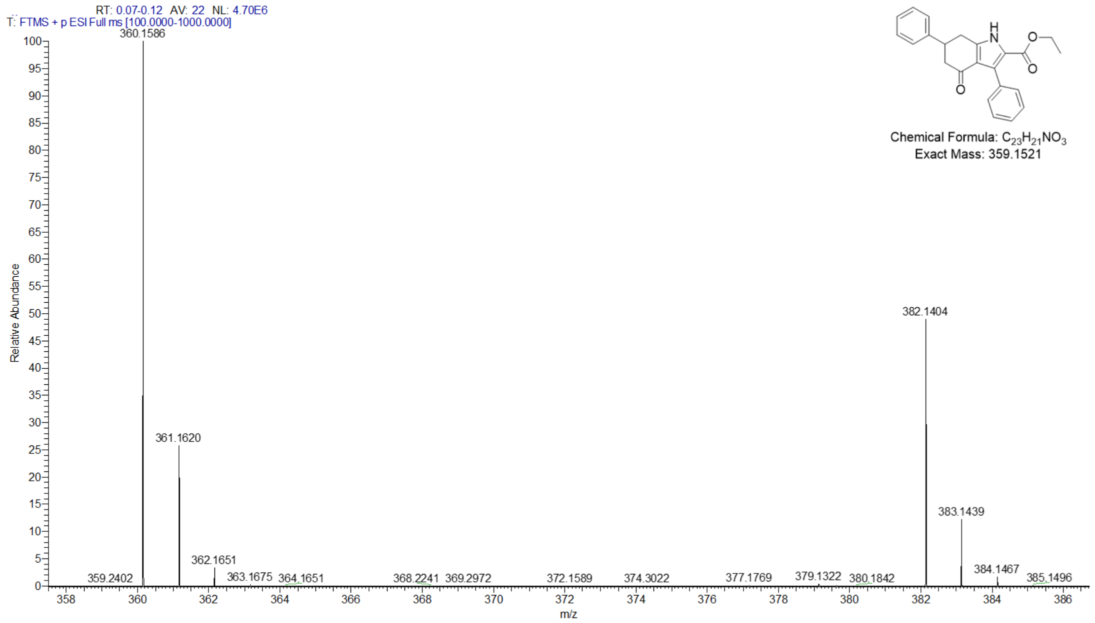

MS spectrum of compound **13**

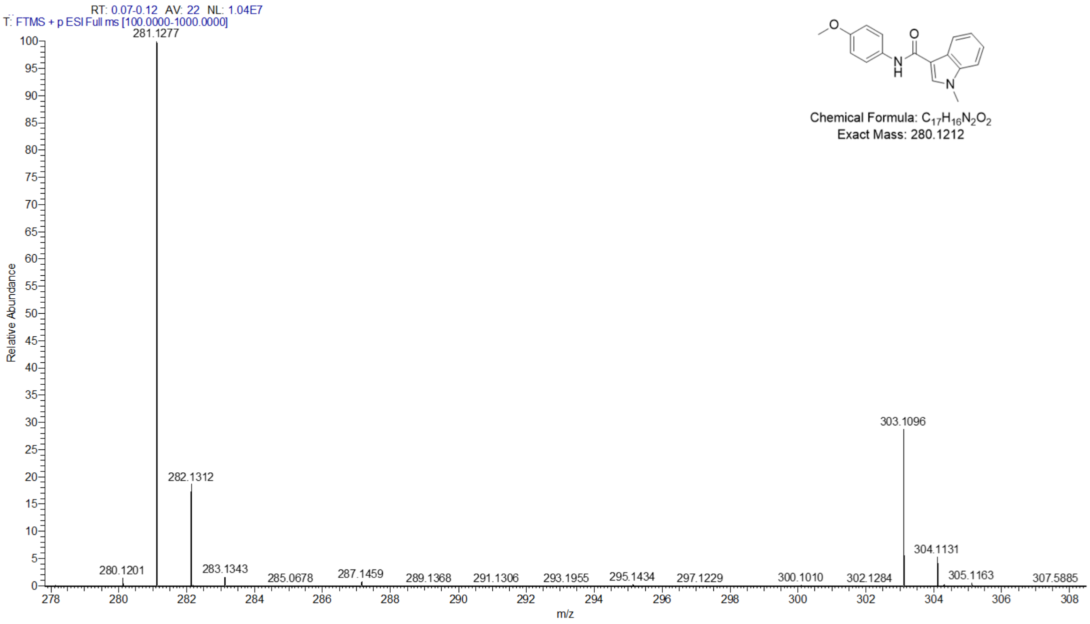

MS spectrum of compound **15**

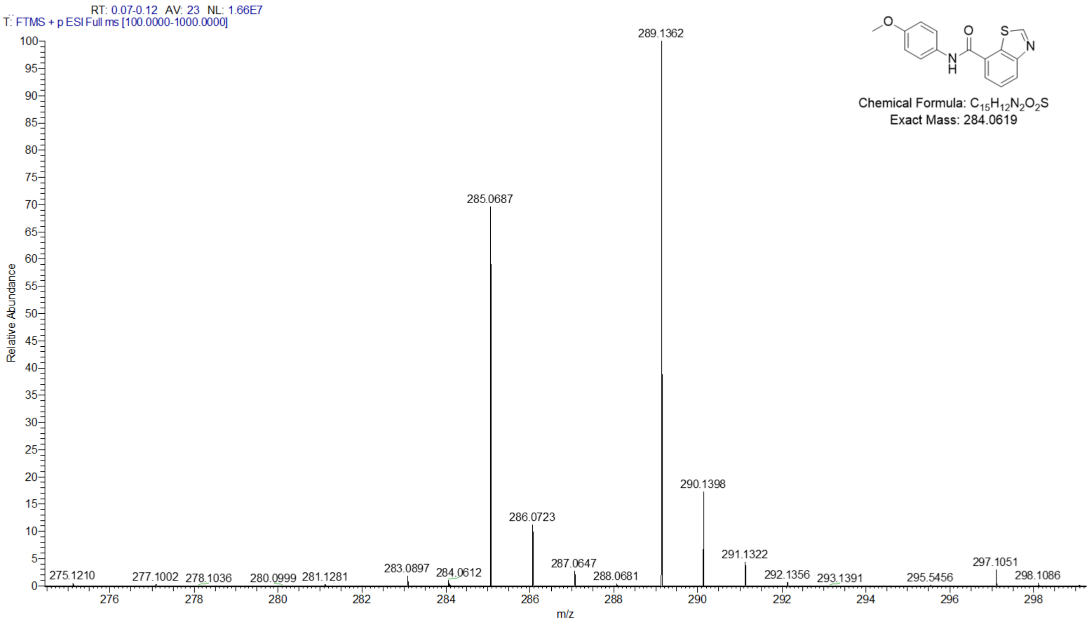

MS spectrum of compound **17**

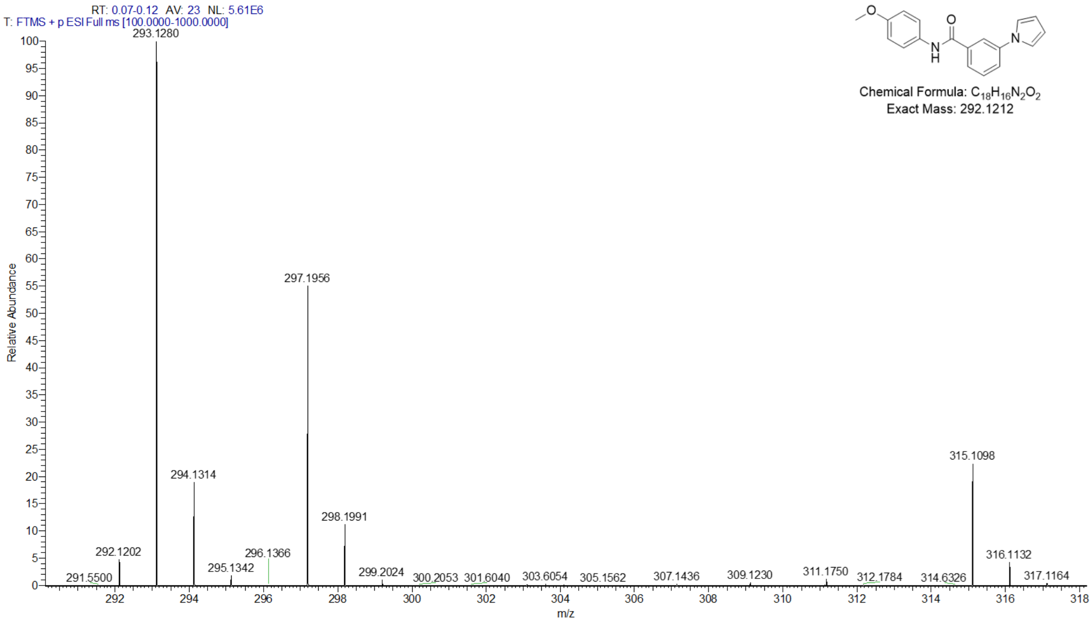

MS spectrum of compound **18**

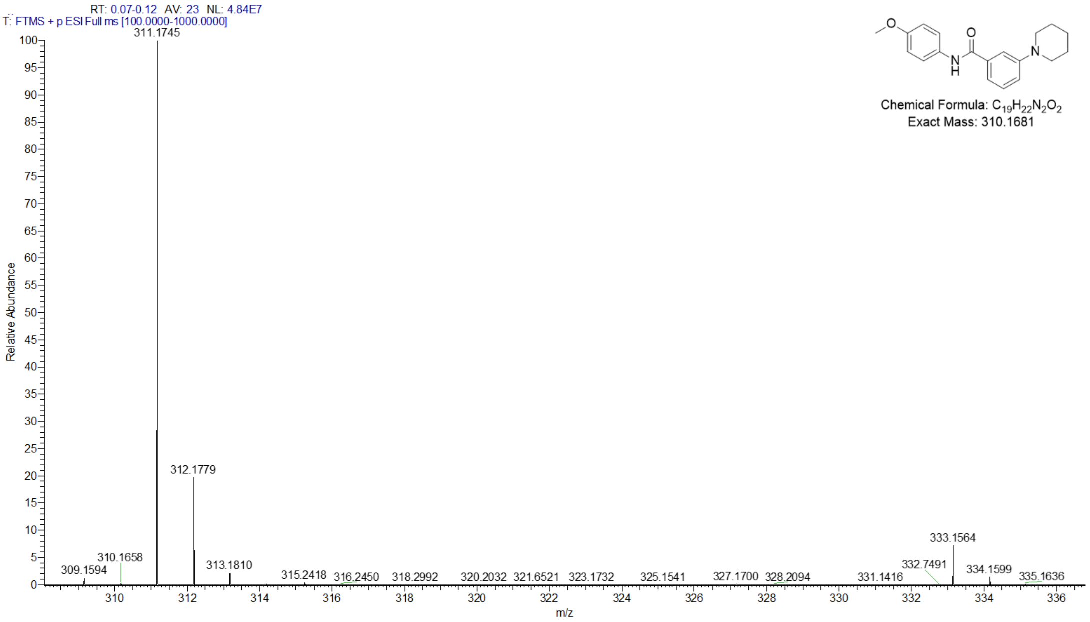

HPLC report annotation

单次进样报告

Agilent

数据文件:	Data File	2025-01-21 17-42-47+08-00-04.dx			
序列名称:	Sequence Name	1260-2025-01-21 16-23-56+08-00			
样品名称:	Sample Name	MA-B			
仪器:	Instrument	1260	进样日期:	Date	2025-01-21 17:43:55+08:00
进样体积:	Injection Volume	10.000 μL	位置:	Location	P2-E3
采集方法:	Acquisition Method	[illegible].amx	类型:	Type	样品
处理方法:	Processing Method	[illegible]out.pmx	样品含量:	Sample content	[illegible]
手动修改:	Manual Modification	无 No			

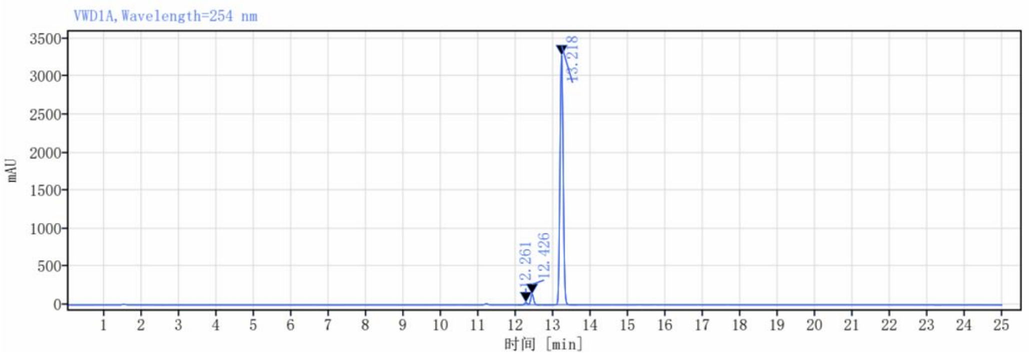

信号: VWD1A, Wavelength=254 nm

Retention time<br>保留时间 [min]	Type<br>类型	Peak width<br>峰宽 [min]	Peak area<br>峰面积	Peak height<br>峰高	Peak area<br>峰面积%	名称
12.261	BV	0.28	152.14	28.26	0.74	
12.426	VB	0.30	750.35	137.56	3.66	
13.218	BB	0.91	19608.60	3273.46	95.60	
		**总和**	**20511.09**			

HPLC purity of compound **8**

单次进样报告

**数据文件:**	2025-01-21 17-42-47+08-00-04.dx		
**序列名称:**	1260-2025-01-21 16-23-56+08-00		
**样品名称:**	MA-B		
**仪器:**	1260	**进样日期:**	2025-01-21 17:43:55+08:00
**进样体积:**	10.000 µL	**位置:**	P2-E3
**采集方法:**	normal.amx	**类型:**	样品
**处理方法:**	*Checkout.pmx	**样品含量:**	0.00
**手动修改:**	无		

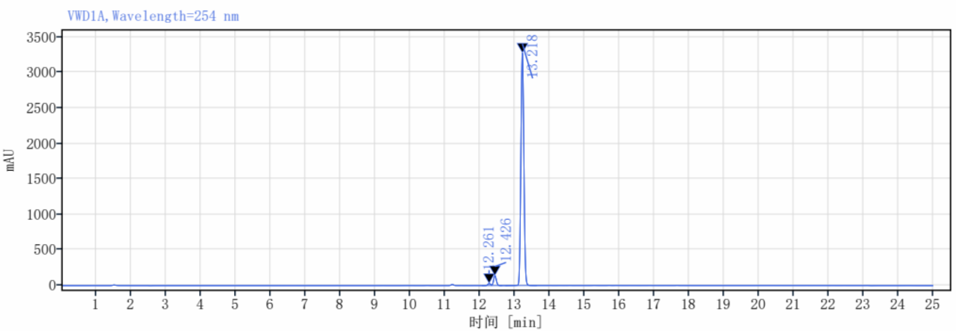

信号: VWD1A, Wavelength=254 nm

保留时间 [min]	类型	峰宽 [min]	峰面积	峰高	峰面积%	名称
12.261	BV	0.28	152.14	28.26	0.74	
12.426	VB	0.30	750.35	137.56	3.66	
13.218	BB	0.91	19608.60	3273.46	95.60	
		**总和**	**20511.09**			

HPLC purity of compound **9**

单次进样报告

Agilent

**数据文件:**	2025-01-21 18-09-02+08-00-05.dx		
**序列名称:**	1260-2025-01-21 16-23-56+08-00		
**样品名称:**	MA-C		
**仪器:**	1260	**进样日期:**	2025-01-21 18:10:10+08:00
**进样体积:**	10.000 µL	**位置:**	P2-E4
**采集方法:**	normal.amx	**类型:**	样品
**处理方法:**	*Checkout.pmx	**样品含量:**	0.00
**手动修改:**	无		

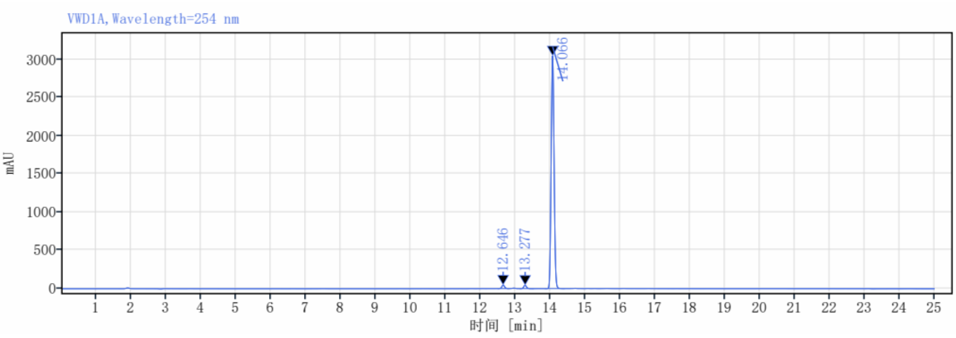

信号: VWD1A,Wavelength=254 nm

保留时间 [min]	类型	峰宽 [min]	峰面积	峰高	峰面积%	名称
12.646	VV	0.33	264.52	45.90	1.44	
13.277	BB	0.41	243.38	43.64	1.33	
14.066	VB	0.56	17825.65	3040.23	97.23	
		**总和**	**18333.55**			

HPLC purity of compound **13**

单次进样报告

**数据文件:**	2025-01-19 23-39-30+08-00-03.dx		
**序列名称:**	1260-2025-01-19 22-46-03+08-00		
**样品名称:**	MA-4		
**仪器:**	1260	**进样日期:**	2025-01-19 23:40:36+08:00
**进样体积:**	1.000 µL	**位置:**	P1-A11
**采集方法:**	normal.amx	**类型:**	样品
**处理方法:**	*Checkout.pmx	**样品含量:**	0.00
**手动修改:**	无		

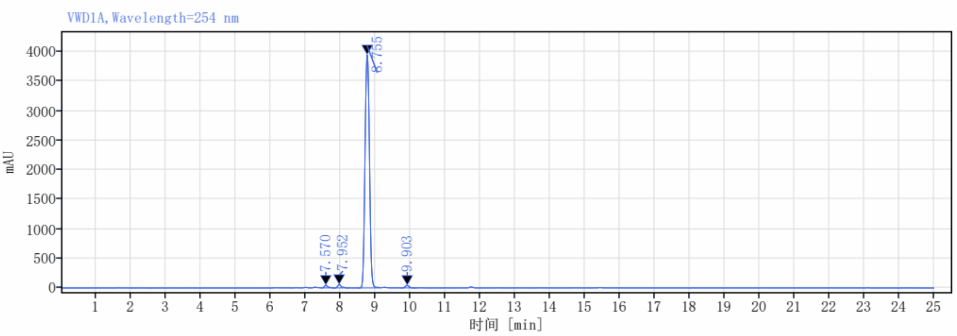

信号: VWD1A,Wavelength=254 nm

保留时间 [min]	类型	峰宽 [min]	峰面积	峰高	峰面积%	名称
7.570	VB	0.37	309.69	45.11	0.86	
7.952	BV	0.40	409.62	58.24	1.14	
8.755	BV	0.67	34761.12	3951.56	97.08	
9.903	BB	0.41	325.84	43.52	0.91	
		**总和**	**35806.28**			

HPLC purity of compound **15**

单次进样报告 Agilent

**数据文件:**	2025-01-16 18-51-02+08-00-06.dx		
**序列名称:**	1260-2025-01-16 16-39-47+08-00		
**样品名称:**	MA-3		
**仪器:**	1260	**进样日期:**	2025-01-16 18:52:06+08:00
**进样体积:**	1.000 µL	**位置:**	P1-F11
**采集方法:**	normal.amx	**类型:**	样品
**处理方法:**	*Checkout.pmx	**样品含量:**	0.00
**手动修改:**	无		

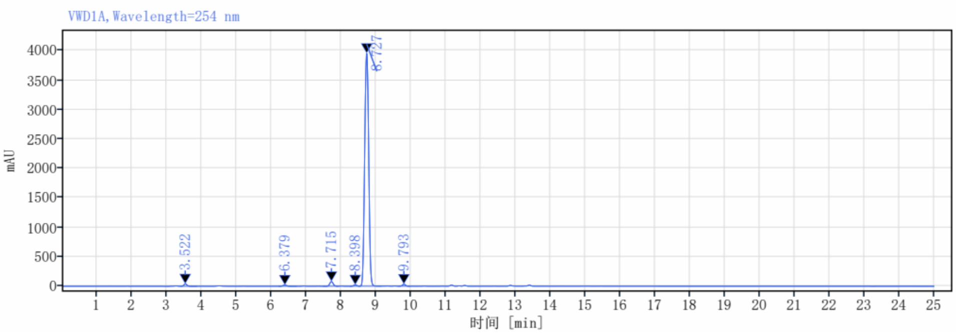

信号: VWD1A,Wavelength=254 nm

保留时间 [min]	类型	峰宽 [min]	峰面积	峰高	峰面积%	名称
3.522	VV	0.46	269.64	39.99	0.82	
6.379	BB	0.52	145.64	19.25	0.44	
7.715	BV	0.43	547.83	79.80	1.67	
8.398	BB	0.28	135.69	21.36	0.41	
8.727	BV	0.62	31497.24	3957.96	96.02	
9.793	BV	0.34	205.38	33.03	0.63	
		**总和**	**32801.43**			

HPLC purity of compound **16**

单次进样报告

Agilent

**数据文件:**	2025-01-16 17-32-21+08-00-03.dx		
**序列名称:**	1260-2025-01-16 16-39-47+08-00		
**样品名称:**	MA-1		
**仪器:**	1260	**进样日期:**	2025-01-16 17:33:27+08:00
**进样体积:**	1.000 µL	**位置:**	P1-F10
**采集方法:**	normal.amx	**类型:**	样品
**处理方法:**	*Checkout.pmx	**样品含量:**	0.00
**手动修改:**	无		

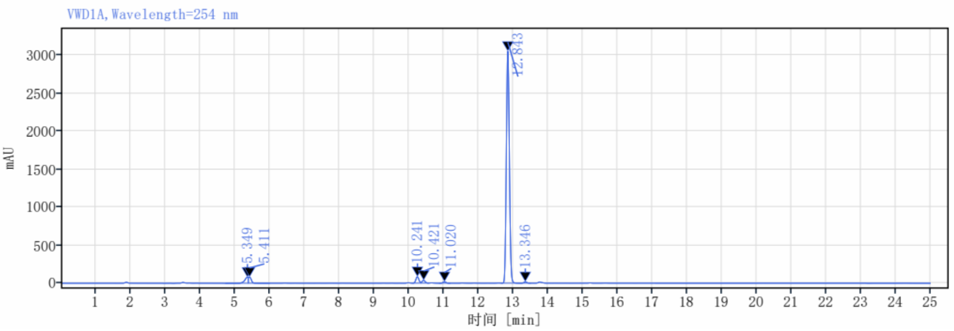

信号: VWD1A,Wavelength=254 nm

保留时间 [min]	类型	峰宽 [min]	峰面积	峰高	峰面积%	名称
5.349	BV	0.66	547.37	78.01	2.68	
5.411	VB	0.62	474.38	79.82	2.32	
10.241	BV	0.24	511.08	84.58	2.50	
10.421	VB	0.22	228.98	37.09	1.12	
11.020	BV	0.33	113.67	18.80	0.56	
12.843	VV	0.57	18439.67	3052.55	90.32	
13.346	VBA	0.28	100.63	17.76	0.49	
		**总和**	**20415.79**			

HPLC purity of compound **17**

单次进样报告

Agilent

**数据文件:**	2025-01-21 19-01-32+08-00-07.dx		
**序列名称:**	1260-2025-01-21 16-23-56+08-00		
**样品名称:**	MA-9		
**仪器:**	1260	**进样日期:**	2025-01-21 19:02:43+08:00
**进样体积:**	1.000 μL	**位置:**	P2-F1
**采集方法:**	normal.amx	**类型:**	样品
**处理方法:**	*Checkout.pmx	**样品含量:**	0.00
**手动修改:**	无		

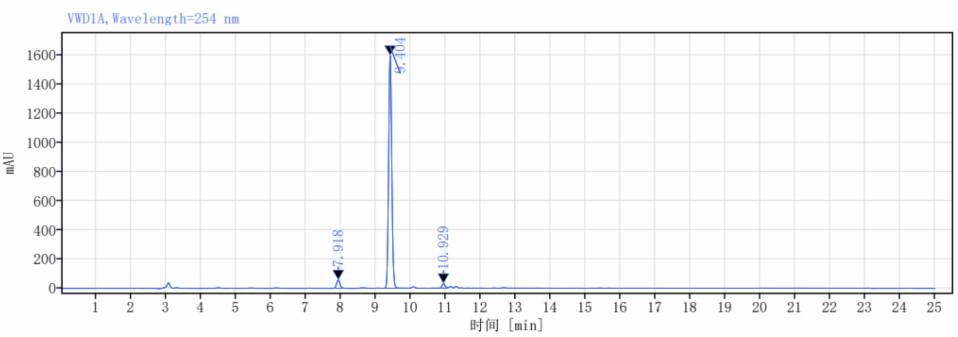

信号: VWD1A, Wavelength=254 nm

**保留时间 [min]**	**类型**	**峰宽 [min]**	**峰面积**	**峰高**	**峰面积%**	**名称**
7.918	BB	0.66	396.13	57.43	3.73	
9.404	BV	0.70	10026.49	1599.64	94.44	
10.929	BV	0.31	194.27	31.89	1.83	
		**总和**	**10616.90**			

HPLC purity of compound **18**

单次进样报告

Agilent

**数据文件:**	2025-01-20 02-42-58+08-00-10.dx		
**序列名称:**	1260-2025-01-19 22-46-03+08-00		
**样品名称:**	MA-7-2		
**仪器:**	1260	**进样日期:**	2025-01-20 02:44:03+08:00
**进样体积:**	5.000 µL	**位置:**	P1-F11
**采集方法:**	normal.amx	**类型:**	样品
**处理方法:**	*Checkout.pmx	**样品含量:**	0.00
**手动修改:**	无		

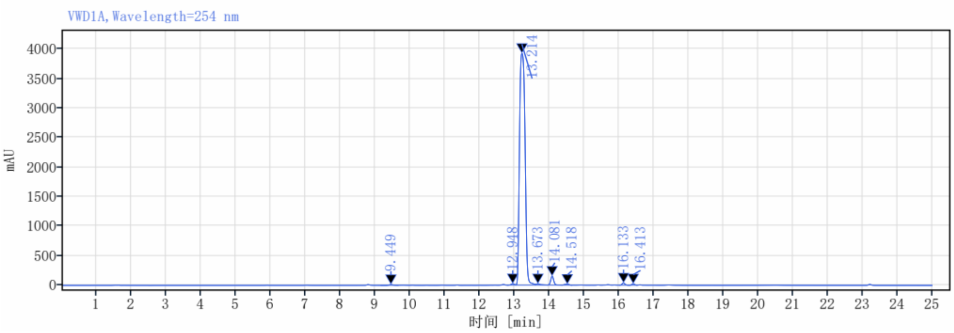

信号: VWD1A,Wavelength=254 nm

保留时间 [min]	类型	峰宽 [min]	峰面积	峰高	峰面积%	名称
9.449	BV	0.51	107.02	13.25	0.24	
12.948	BV	0.25	155.74	24.55	0.34	
13.214	VV	0.56	43381.94	3926.28	95.79	
13.673	VV	0.33	215.00	23.53	0.47	
14.081	VV	0.42	945.42	152.13	2.09	
14.518	VB	0.29	139.63	15.69	0.31	
16.133	VB	0.28	215.22	37.49	0.48	
16.413	BV	0.23	129.95	23.45	0.29	
		**总和**	**45289.91**			
